# Methamphetamine hijacks chaperone-mediated autophagy to degrade GPX4, driving ferroptosis-precipitated cognitive decline and addictive pathogenesis

**DOI:** 10.1186/s40478-026-02342-7

**Published:** 2026-06-15

**Authors:** Zizhen Si, Dan Zhu, Jianan Lv, Yang Shen, Xidi Wang, Jiahui Zhou, Zhiting Zou, Wenting Wei, Longhui Li, Xing Xu, Yaping Chen, Wenhua Zhou, Yu Liu

**Affiliations:** 1 Department of Psychiatry, Zhejiang Key Laboratory of Drug Addiction and Brain Health, The Affiliated Kangning Hospital of Ningbo University, Ningbo, China; 2 Health Science Center, Ningbo University, 818 Fenghua Road Jiangbei Dist, Ningbo, 315211 Zhejiang P.R. China; 3 Department of Psychology, Collage of Teacher Education, Ningbo University, Ningbo, China; 4 School of Material Science and Chemical Engineering, Ningbo University, Ningbo, China; 5 Zhejiang Rongjun Rehabilitation Hospital, Ningbo Psychiatric Hospital, Ningbo, China; 6 Addiction treatment laboratory of Zhejiang, Hangzhou, China

**Keywords:** Methamphetamine, Self-administration, Addictive Pathogenesis, Chaperone-mediated autophagy, Ferroptosis

## Abstract

Methamphetamine (METH) addiction is associated with progressive cognitive decline and maladaptive behaviors, but the molecular mechanisms bridging proteostatic dysfunction to neural circuit degeneration remain poorly defined. We hypothesized that METH hijacks chaperone-mediated autophagy (CMA), a lysosomal quality-control pathway, to drive neurodegeneration through ferroptosis. Using chronic METH self-administration models, hippocampal neurons, and CMA-targeted approaches, we demonstrated that METH coerces CMA components (HSC70-LAMP2A) to recognize a non-canonical ^124^NVKFD^128^ degron on glutathione peroxidase 4 (GPX4), resulting in its lysosomal degradation. This CMA-dependent GPX4 depletion disrupted glutathione recycling, unleashing lethal lipid peroxidation and hippocampal ferroptosis. Critically, neuronal CMA ablation rescued spatial memory deficits and reduced compulsive drug-seeking behaviors, establishing CMA hyperactivity as a causal driver of addiction-related neuropathology. Our findings extend the oxidative stress-centric model of METH toxicity by revealing CMA as a pathological switch that converts physiological proteostasis into a self-destructive cascade. The CMA-GPX4 axis, mechanistically linking protein quality control failure, iron-dependent cell death, and behavioral dysfunction, represents a druggable target, with CMA inhibitors showing preclinical efficacy in mitigating METH-induced neuropsychiatric deterioration. By redefining addiction-associated neurodegeneration as a disorder of hijacked proteostasis, this work provides a unified framework for targeting shared mechanisms in substance use disorders and neurodegenerative diseases.

**Graphical abstract:**

**Figure:**
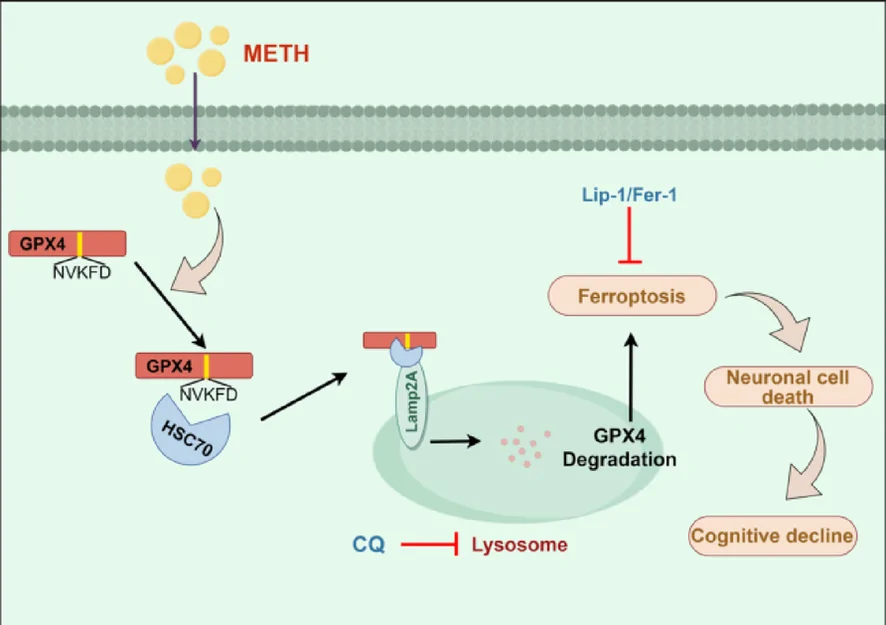

**Supplementary Information:**

The online version contains supplementary material available at 10.1186/s40478-026-02342-7.

## Introduction

Methamphetamine (METH) abuse represents a major public health crisis, with its highly addictive properties well-documented in relation to dysregulated monoaminergic neurotransmission and drug-context memory formation [1, 2]. Beyond these established mechanisms, emerging clinical evidence reveals that chronic METH exposure induces progressive cognitive deterioration extending beyond addiction-related behaviors [3, 4, 5]. These non-drug-specific cognitive impairments frequently persist during abstinence and correlate strongly with relapse vulnerability [6, 7], yet their underlying neuropathology remains poorly defined.

Current therapeutic approaches for substance use disorders, including cognitive remediation therapies and glutamatergic modulation [8, 9, 10], demonstrate limited efficacy in METH addiction, partly due to insufficient targeting of upstream neurodegenerative processes [8, 11]. While synaptic remodeling in drug-associated memory consolidation has been extensively characterized [9, 12, 13], the molecular mechanisms bridging proteostatic dysfunction to neural circuit degeneration in addiction-associated cognitive decline remain largely unexplored.

Recent advances have implicated iron-mediated oxidative damage in various neuropsychiatric disorders [14, 15], but whether programmed cell death mechanisms—particularly ferroptosis, an iron-dependent form of regulated cell death driven by lipid peroxidation—contribute to METH-induced cognitive pathology remains unknown. CMA, a selective lysosomal degradation pathway for proteins containing KFERQ-like motifs [16, 17], represents a critical nexus between protein quality control and redox homeostasis. In neurodegenerative contexts, CMA serves dual roles: while essential for removing misfolded protein aggregates in Alzheimer’s disease models [18], its aberrant activation can paradoxically deplete key antioxidant defenses, including GPX4—the central regulator of ferroptosis [19, 20].

This duality raises a fundamental question: could METH-induced cognitive decline involve the exploitation of CMA’s quality control machinery to trigger ferroptotic neurodegeneration? Current models of addiction-related neurotoxicity predominantly focus on macroautophagy [21] or ubiquitin-proteasome systems [22], overlooking CMA’s specialized capacity for regulating redox-sensitive substrates. The potential intersection between CMA dysregulation, GPX4 stability, and hippocampal neuronal survival in the context of METH addiction represents a significant knowledge gap with implications for understanding both substances use disorders and neurodegenerative processes.

To specifically investigate the neurobiological mechanisms underlying voluntary drug intake and addiction-like behaviors, we employed an intravenous METH self-administration (SA) paradigm. In this model, rodents are surgically implanted with a chronic catheter connected to the jugular vein, allowing them to voluntarily press a lever to receive precise intravenous METH infusions. This method is distinct from passive injection technique as it directly models the volitional aspect of human drug seeking and taking, making it a highly relevant model for studying addiction pathogenesis.

Here, we investigate whether METH hijacks the CMA pathway to drive hippocampal neurodegeneration through ferroptosis. By integrating behavioral analyses with molecular approaches in complementary in vivo and in vitro models, we test the hypothesis that METH subverts CMA to degrade GPX4, thereby establishing a novel “proteostasis-redox” axis in addiction pathophysiology and identifying potential therapeutic targets for mitigating METH-induced cognitive comorbidities.

## Materials and methods

### Reagents and resources

All key reagents used in this study are summarized below for clarity. A comprehensive list of pharmacological compounds, including sources and catalog numbers, is provided in Table S1. Detailed information on specific antibodies and assay kits is integrated within the respective methodological subsections that follow. Antibodies: All primary and secondary antibodies are described in the *Western Blot Analysis* subsection. Assay Kits: Commercial kits are detailed in the corresponding *Biochemical Assays* subsection.

## Participants

This study enrolled 425 participants between December 2020 and November 2022. Inclusion criteria were: (1) meeting diagnostic criteria for substance use disorder (SUD); (2) age between 18 and 65 years; (3) ability to provide written informed consent. The exclusion criteria were as follows: patients with severe SUD, severe mental illnesses (e.g., schizophrenia, bipolar disorder), severe physical diseases, and an inability to consent or maintain contact. Of the 425 participants, 254 completed the study. The study was approved by the Ethics Committee of the Affiliated Hospital of Ningbo University School of Medicine, and all participants provided written informed consent (Clinical trials registration number: ChiCTR2100049818).

## Human cognitive assessment

Cognitive function in all human participants was assessed using the Montreal Cognitive Assessment (MoCA; version 7.1) and the Mini-Mental State Examination (MMSE). Both tests provide a global cognitive score (range 0–30), with a score below 26 on the MoCA indicating potential cognitive impairment. These instruments capture several cognitive domains, with a primary focus on those sensitive to medial temporal lobe and hippocampal function, notably episodic memory. This was assessed by the delayed recall of words (3 words in MMSE; 5 words in MoCA). All assessments were administered by trained personnel in a quiet room.

## Experimental animals

Male C57B/L6 mice (6–8 weeks old) were purchased from Beijing Vital River Laboratory Animal Technology Co., Ltd. Lamp2a^flox/flox^ and CaMKIIa-Cre mice were provided by Shanghai model organisms. CaMKIIa-Cre mice were mated with Lamp2a^flox/flox^ mice to generate a neuronal-specific CMA-deficient mouse model (Lamp2a-CKO mice). All mice were bred in the Experimental Animal Central of Ningbo University, housed under a 12 h light/dark cycle (8:00 a.m. to 8:00 p.m.) with controlled humidity (40%–60%) and temperature (23 ± 1 ℃). For METH injection, mice were intraperitoneally injected with METH (5 mg/kg) once daily for seven consecutive days. For ferroptosis inhibition, C57B/L6 mice received intranasal injection of liproxstatin-1 (Lip-1, 5 mg/kg, MCE, USA, Catalog HY12726) or ferrostatin-1 (Fer-1, 5 mg/kg, MCE, USA, Catalog HY-100579) once daily from the day of METH exposure until the mice were sacrificed. For CMA inhibition, C57B/L6 mice received intranasal injection of chloroquine (CQ, 20 mg/kg, MCE, USA, Catalog HY-17589 A) once daily from the day of METH exposure until the mice were sacrificed. All animal procedures were conducted in accordance with the Guide for the Care and Use of Laboratory Animals from the National Institutes of Health and were approved by the Institutional Animal Care and Use Committee of Ningbo University.

## Surgery and METH SA training

METH (> 90% purity) was obtained from the Ningbo Anti-drug Bureau of the Ningbo Police and dissolved in 0.9% saline. The procedures for surgery and METH SA training were conducted as previously described [23]. The mice were anesthetized with 1.0% sodium pentobarbital and implanted with a silastic catheter in the jugular vein, connected to a cannula subcutaneously to a polyethylene assembly on the back of the mice. After seven days of recovery, the mice underwent 14-day METH SA training. Daily fixed-ratio 1 (FR1) training lasted 4 h, or until 200 METH infusions (0.05 mg/kg/infusion) were reached in one session. Stable METH SA was defined as ≥ 25 METH infusions, an active/inactive response ratio > 2:1 for three consecutive days, and < 20% variability in daily METH infusions across 3 straight sessions. All in vivo METH exposure in this study was achieved through this SA protocol.

### Behavioral tests

Following the establishment of stable METH SA, cognitive functions were assessed using the Morris water maze (MWM), Y-maze, and novel object recognition (NOR) tests. All behavioral tests were conducted during the light phase in dedicated, sound-attenuated rooms.

## MWM

A circular pool was filled with milk-tinted water maintained at 25 °C. A platform was hidden below the water surface in one pool quadrant. Prior to the formal acquisition phase, mice were given a single 60 s habituation trial in the pool without the platform to reduce stress and neophobia. Mice were trained in four trials daily for five consecutive days, starting at different positions for each trial, and allowed to swim freely for 90 s. The time mice spent swimming to the platform was recorded as escape latency; however, if a mouse failed to find the platform within 90 s, the researcher directed and kept it on the platform for 15 s. Probe trials were performed after the last trial of the acquisition period, and the platform was removed. The mice were allowed to swim freely for 90 s. The time spent in the target quadrant that previously contained the platform, and the number of platform location crosses were recorded.

## Y-maze spontaneous alternation test

Spatial working memory was assessed using the Y-maze test. Mice were placed in the center of a symmetrical Y-shaped maze and allowed to explore freely for 8 min. No prior training or habituation session was conducted for this test, as it relies on the innate exploratory behavior of mice to assess spontaneous alternation, a measure of spatial working memory. The apparatus consisted of three identical opaque arms (40 cm long × 15 cm wide × 20 cm high) symmetrically arranged at 120° angles. Mice were placed at the center of the maze and allowed to explore freely for 8 min under dim light. Sessions were recorded, and arm entries were manually scored by an experimenter blinded to the experimental groups. An arm entry was defined as all four paws entering an arm. The percentage of spontaneous alternation was calculated using the formula:

$$\begin{aligned} \% Alternation = & \left[ {Number\;of\;Alternations/} \right. \\ & \left. {(Total\;Arm\;Entries - 2)} \right] \\ \end{aligned} $$

where an “alternation” was defined as three consecutive entries into three different arms. For example, the sequence A→B→C represents one alternation. Total arm entries and alternation behavior were analyzed, with fewer alternations interpreted as a deficit in spatial working memory.

### NOR

On Day 1 (Habituation), mice were allowed to freely explore the empty arena for 10 min. On Day 2 (Familiarization), two identical objects were placed in the arena, and mice explored for 10 min. On Day 3 (Test), conducted 24 h later, one familiar object was replaced with a novel object, and mice explored for 10 min. The time spent exploring each object was recorded. The recognition index (RI) = time spent exploring a new object/time spent exploring a new object and the last identical object × 100%. Preference index = time spent exploring one of the identical objects/time spent exploring the two identical objects × 100%.

### Inclusion and exclusion criteria

Mice were included in the final behavioral and molecular analyses only if they successfully met the predefined criteria for stable METH SA (as described above). Mice were excluded from the study if they exhibited any of the following: severe postoperative complications (e.g., infection, weight loss > 20%), catheter failure during the SA training period, or if they were identified as extreme outliers in behavioral performance (e.g., floating in MWM, complete lack of exploration in NOR/Y-maze) based on pre-established criteria.

### Cell culture

HT22 cells were cultured in Dulbecco’s Modified Eagle’s Medium (DMEM) with 10% FBS (Gibco, Cat# 10099141 C, USA) and 100 units/mL penicillin/streptomycin. Hippocampal-derived primary neurons were isolated as previously described [24]. After isolation, primary neurons were plated on poly-D-lysine-coated (Thermo Scientific™, Cat#A3890401, USA) plates and maintained in Neurobasual Plus medium supplemented with B-27 Plus (Thermo Scientific™, Cat# A3653401, USA) and GlutaMAX (Thermo Scientific™, Cat#35050061, USA). Cultures were kept in a humidified incubator at 37 °C with 5% CO₂. Neurons were cultured for 12–14 days in vitro (DIV) to allow for full maturation prior to any experimental manipulations. At DIV 12–14, cultures were treated with METH (1 or 2 mmol/L) or vehicle for 24 h.

### Drug treatment and experimental groups

All chemical reagents were obtained from commercial sources. N-Acetylcysteine (NAC), Chloroquine (CQ), 3-Methyladenine (3-MA), MG-132, and PS-341 were purchased from MedChemExpress (USA). E64d was obtained from Santa Cruz Biotechnology (USA). Pepstatin A was purchased from Merck KGaA (Germany). A GPX4 Activity Detection Kit was sourced from Elabscience Biotechnology (China). Detailed information on each compound is provided in Table S1.

### Experimental design

HT22 cells or primary hippocampal neurons were seeded and cultured to the appropriate confluence. To investigate the role of specific pathways in METH-induced effects, cells were divided into the following key treatment groups:

1. **Vehicle Group**: Treated with vehicle.
2. **Control Group**: No Treatment.
3. **METH Group**: Treated with METH alone.
4. **Inhibitor + METH Groups**: Pre-treated with a specific pharmacological inhibitor (as listed in Table S1) for 1 h, followed by co-exposure to the inhibitor and METH for the designated treatment period. These groups were used to determine if blocking a pathway could rescue the METH-induced phenotype.

For all experiments, cells were harvested at the indicated time points post-treatment for subsequent biochemical analyses.

### Cell viability assays and measurement of glutathione (GSH) and malondialdehyde (MDA) levels

Cell viability was determined using the MTS assay (Promega, Cat# G3581, USA). Total GSH was measured in tissue or cell lysates using a GSH detection kit (Promega, Cat# V6911, USA). MDA levels were measured in tissue or cell lysates using an MDA detection kit (Beyotime Technology, Cat# S0131, China). All procedures were conducted according to the instructions of the manufacturer.

### DAB-enhanced Perl’s Prussian blue iron staining, Iron detection and Lysosome Isolation

Iron deposition levels were stained using a DAB kit (Zsbio, Cat# ZLI-9018, China). Lysosome fractions were isolated using a Lysosome Enrichment Kit (Pierce, Cat# 89839, USA).

To visualize histologically detectable iron (primarily Fe³⁺) deposition in hippocampal tissue, Perls’ Prussian blue staining with DAB enhancement was performed on paraffin-embedded Sect.  (5 μm). Briefly, after deparaffinization and rehydration, sections were incubated in a freshly prepared Perls’ working solution (3% potassium ferrocyanide and 3% hydrochloric acid, 1:1 v/v) for 30 min at room temperature. Following thorough washing, the Prussian blue complex was enhanced using a commercial DAB chromogen kit (Zsbio, Cat# ZLI-9018) according to the manufacturer’s instructions to generate a brown precipitate. Sections were then counterstained with Nuclear Fast Red, dehydrated, cleared, and mounted. Stained sections were imaged under bright-field microscopy (Nikon Eclipse Ti2). The area of DAB-positive iron deposition in the hippocampal CA1 region was quantified in at least three non-overlapping fields per section using ImageJ software (NIH) and expressed as the percentage of positive area relative to the total region of interest.

The labile iron pool (LIP), primarily reflecting redox-active Fe²⁺, was quantified using an Iron Assay Kit (Colorimetric, ab83366, Abcam). Briefly, Fe²⁺ level was determined directly by chelation with a chromogen. Total iron (Fe²⁺ + Fe³⁺) was measured in parallel after reduction of all iron to the Fe²⁺ state. The level of Fe³⁺ was calculated by subtracting Fe²⁺ from total iron. All values were normalized to total protein concentration. To validate the specificity and sensitivity of the iron assay, HT22 cells were treated with ferric ammonium citrate (FAC, HY-B1645, MedChemExpress, USA, 100 µM, 24 h) as a positive control for iron loading, or deferoxamine (DFO, HY-B1625, MedChemExpress, USA, 100 µM, with 6 h pre-treatment followed by 18 h co-incubation) as a negative control for iron chelation. The concentrations were chosen based on preliminary viability assays and established protocols in neuronal ferroptosis research. To validate the specificity of the Iron detection kit, we performed an additional method-control experiment, which includes Positive (FAC) and Negative (DFO) controls. This experiment confirms that the probe’s signal is indeed responsive to and specific for changes in the labile iron pool.

### KFERQ-PA-mCherry reporter assay

The pLVX-KFERQ-PA-mCherry plasmid was purchased from Sangon Biotechnology. After being infected with pLVX-KFERQ-PA-mCherry, lentivirus HT22 cells were photoactivated using a 405/20-nm LED array. The HT22 cells were subsequently fixed and co-stained with DAPI. A Leica TCS SP8 confocal microscope was used to capture the images.

### Fluorescence-activated cell sorting (FACS) assays

Calcein-AM (50 nM, MedChemExpress, Cat# HY-D0041, USA) was utilized to determine cell viability. HT22 cells or hippocampal-derived primary neurons were trypsinized, washed twice, and stained with calcein-AM for 30 min at 37 °C. After washing with PBS twice, the samples were analyzed immediately using flow cytometry (BECKMAN CytoFlex S, USA).

### ROS measurement

ROS level in hippocampal-derived primary neurons and HT22 cells was determined by a Cell Meter Fluorimetric Intracellular Total ROS Activity Assay Kit (AAT Bioquest, Cat# 22900, USA) and C11-BODIPY Kit (Invitrogen, Cat# D3861, USA) that can be used for study lipid peroxidation and antioxidant properties in living cells. The fluorescence of Total ROS Activity Assay Kit was measured using a fluorescence microplate reader (FLx800, BioTek, USA) at 650 nm of excitation wavelength. The cells were imaged using a laserscanning confocal microscope (Leica TCS SP8, Germany). The samples satined with C11-BODIPY Kit were analyzed using flow cytometry (BECKMAN CytoFlex S, USA).

### Western blotting and immunoprecipitation

Western blotting and immunoprecipitation were conducted as previously described [25]. The primary antibodies used in this study included HSC70 (1:2000, Proteintech, Cat# 10654-1-AP, China), FSP1 (ferroptosis suppressor protein 1, 1:4000, Proteintech, Cat# 20886-1-AP, China) and β-actin (1:10000, Proteintech, Cat# 66009-1-Ig, China) were purchased from Proteintech. GPX4 (1:1000, abcam, Cat# ab125066, England) was purchased from Abcam. LAMP-2a (1:1000, Invitrogen, Cat# 51-2200, USA) from Invitrogen.

### Immunocytochemistry and co-localization analysis

#### Immunofluorescence staining

HT22 cells were cultured under experimental conditions on glass coverslips. Following treatment, cells were fixed with 4% paraformaldehyde in PBS for 10 min at 37 °C, then permeabilized with 0.05% Triton X-100 in PBS for 20 min at room temperature. After permeabilization, cells were blocked with 2% normal goat serum (Product # 31872, Thermo Fisher Scientific, USA) in PBS for 30 min at room temperature to prevent non-specific binding.

Cells were subsequently incubated with the following primary antibodies diluted in blocking buffer: Rabbit recombinant monoclonal anti-GPX4 antibody (1:50 dilution; Product # MA5-32827, Thermo Fisher Scientific, USA) overnight at 4 °C. Rat monoclonal anti-LAMP2 antibody (10 µg/mL; Product # MA1-165, Thermo Fisher Scientific, USA) for 1 h at room temperature. After primary antibody incubation, cells were washed three times with PBS and incubated with the following fluorescently-labeled secondary antibodies for 1 h at room temperature in the dark: DyLight Cy3-conjugated goat anti-rat IgG (1:1000 dilution; Product # ab6953, Abcam, USA; excitation/emission: ~550/570 nm); Alexa Fluor 594-conjugated goat anti-rabbit IgG (1:1000 dilution; Product # JX00101139, Jackson ImmunoResearch, USA; excitation/emission: ~590/617 nm). Images were captured using a Leica SP8 confocal microscope equipped with filter sets for Cy3 (excitation 552 nm, emission 565–605 nm) and Alexa Fluor 594 (excitation 594 nm, emission 600–630 nm). Coverslips were mounted onto glass slides using anti-fade mounting medium.

#### Image acquisition and co-localization analysis

Confocal images were acquired using a confocal laser scanning microscope (Leica TCS SP8, Germany) equipped with a 63× oil immersion objective. Image capture parameters were kept consistent across all experimental conditions to enable quantitative comparisons.

Co-localization analysis was performed using ImageJ with JACoP plugin. Following background subtraction, the Pearson’s correlation coefficient was calculated for the GPX4 and LAMP2 channels from at least 20 cells across three independent experiments. Threshold values were determined automatically using the software’s built-in algorithm. Statistical analysis of co-localization data was performed using SPSS (version 20.0; IBM, USA).

### Quantitative real time polymerase chain reaction (qPCR)

Total RNA was extracted using TRIzol (Invitrogen, Cat# 15596018, USA) and reverse transcribed using the PrimeScript RT reagent kit (Takara, Cat# RR036, Japan). qRT-PCR was performed on an ABI StepOne Plus using SYBR Green^®^ Premix Ex Taq (Takara, Cat# RR820, Japan) as described previously [25]. The relative gene expression level was normalized to GAPDH and calculated using the 2^−ΔΔCT^ method.

#### Statistics

Statistical Package for the Social Sciences software (version 20.0) (SPSS, USA) was utilized for data analysis. Data are presented as the mean ± standard deviation (SD). Statistical analysis was performed, and graphs (histograms) were generated using GraphPad Prism (version 9.0) software. A one- or two-way analysis of variance with multiple comparisons was performed with Tukey post hoc correction. Figure legends show the details of the statistical analysis and sample size. Statistical significance was considered at *P* < 0.05, **P < 0.01, and ***P < 0.001. All data analyses were repeated at least thrice.

## Results

### METH induces conserved hippocampal-dependent cognitive rigidity across species

To investigate the impact of METH on cognitive function, we first conducted a clinical cohort study followed by a series of validated behavioral tests in a preclinical model. Clinical Cohort Analysis. This study initially enrolled 425 participants, with 254 individuals completing the protocol. Among these, 146 were diagnosed as active METH users or individuals undergoing rehabilitation for METH use disorder, while 108 served as age- and gender-matched healthy controls without a history of METH use (Fig. S1A-C). Cognitive performance, encompassing general learning and memory, was assessed using MMSE and MoCA. Comparative analysis revealed a statistically significant reduction in both MMSE and MoCA scores in the METH user group relative to controls, demonstrating substantial cognitive impairment associated with METH exposure in humans (Fig. S1D, E). Critically, analysis of the hippocampal-sensitive memory sub-scores revealed a profound deficit (Fig. S1F, G), strongly supporting the link between METH use, hippocampal dysfunction, and cognitive decline.

**Fig. 1:**
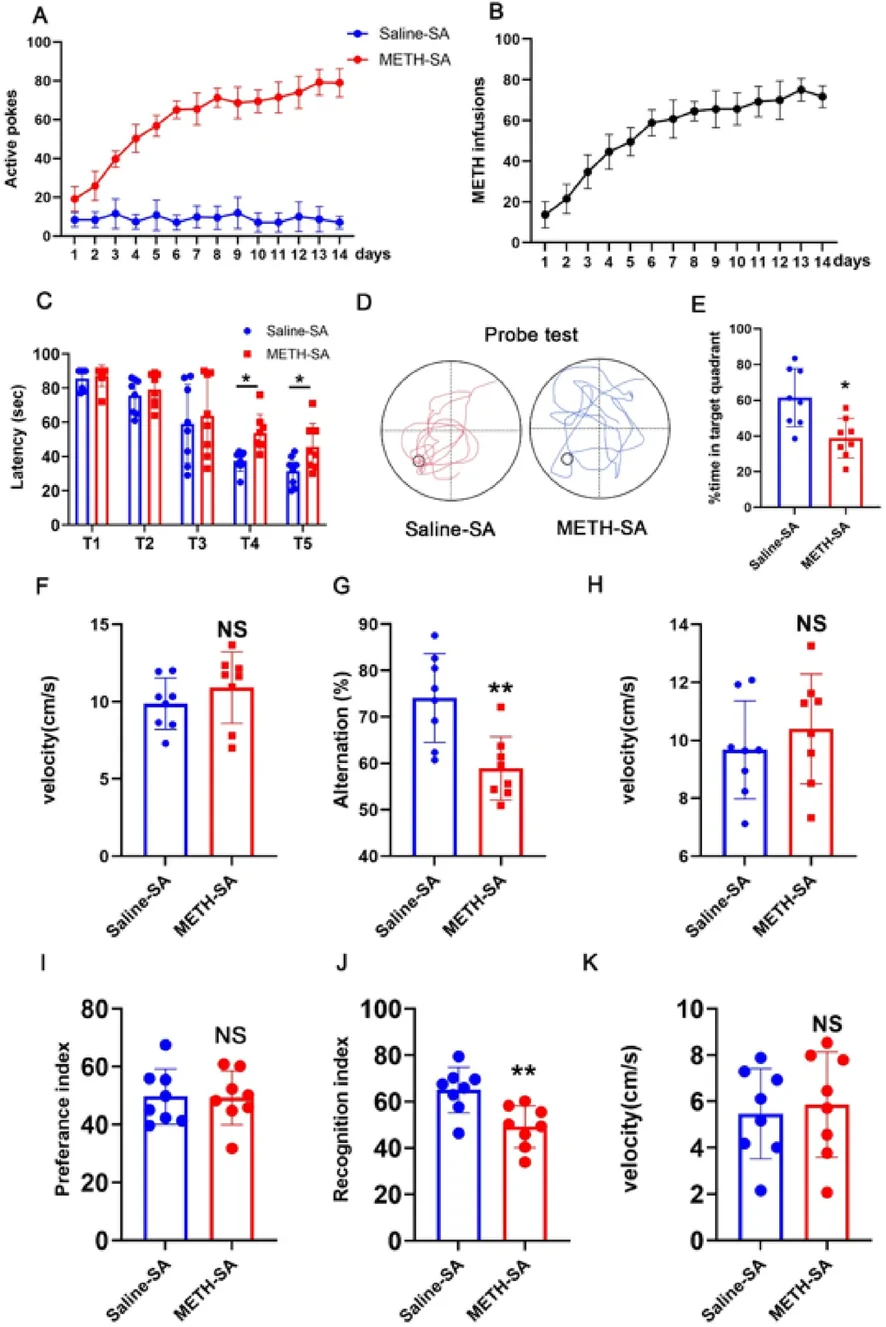
Cognitive decline in METH SA mice. **A** METH-reinforced active nose-pokes. *N* = 10 mice/group. **B** METH-reinforced infusions. *N* = 10 mice/group. **C** Escape latencies (s) of saline-SA and METH SA mice in the MWM test. **D** Swim paths, **E** percentage of time spent swimming in the target quadrant, and **F** average velocity during the probe test for mice in the MWM test. **G** Alternation of arms in the spontaneous alternation test of mice in the Y-maze test and **H** average velocity during the spontaneous alternation test of mice in the Y-maze test. NOR. Preference index (I), recognition index **J**, and **K** average velocity during the NOR test. For all figures: Values mean ± SD. One-way ANOVA or two‐way ANOVA. *N* = 8 mice/group. *P < 0.05, **P < 0.01, and ***P < 0.001

Preclinical Behavioral Assessment. To establish a translational model, C57BL/6 mice underwent a 14-day METH SA or saline-SA protocol. The METH SA cohort successfully acquired operant responding, rapidly learning to discriminate between active and inactive nose-pokes and establishing stable SA behavior (Fig. 1A, B). Subsequent behavioral phenotyping of these mice was performed to evaluate hippocampal-dependent cognitive domains. In the MWM test for spatial reference memory, METH SA mice exhibited significantly prolonged escape latencies during the final two training days compared to saline-SA controls (Fig. 1C). This deficit was corroborated during the probe trial, where METH SA mice spent significantly less time exploring the target quadrant (Fig. 1D, E). Working memory, assessed via the Y-maze spontaneous alternation test, was also compromised. METH SA mice demonstrated a significantly lower rate of spontaneous alternation than controls, indicating a pronounced working memory deficit (Fig. 1G, H). Finally, recognition memory was evaluated using the NOR test. While all groups showed similar exploration during the familiarization phase (Fig. 1I), METH SA mice exhibited a significantly lower recognition index in the test phase compared to saline-SA controls (Fig. 1J, K), confirming impaired NOR. Collectively, these convergent findings from clinical and preclinical studies demonstrate that METH exposure induces significant and conserved deficits in multiple domains of learning and memory, including spatial, working, and recognition memory, thereby establishing a cross-species phenotype of hippocampal-dependent cognitive impairment.

### Intrinsic vulnerability of hippocampal neurons to METH-induced ferroptosis

**Fig. 2:**
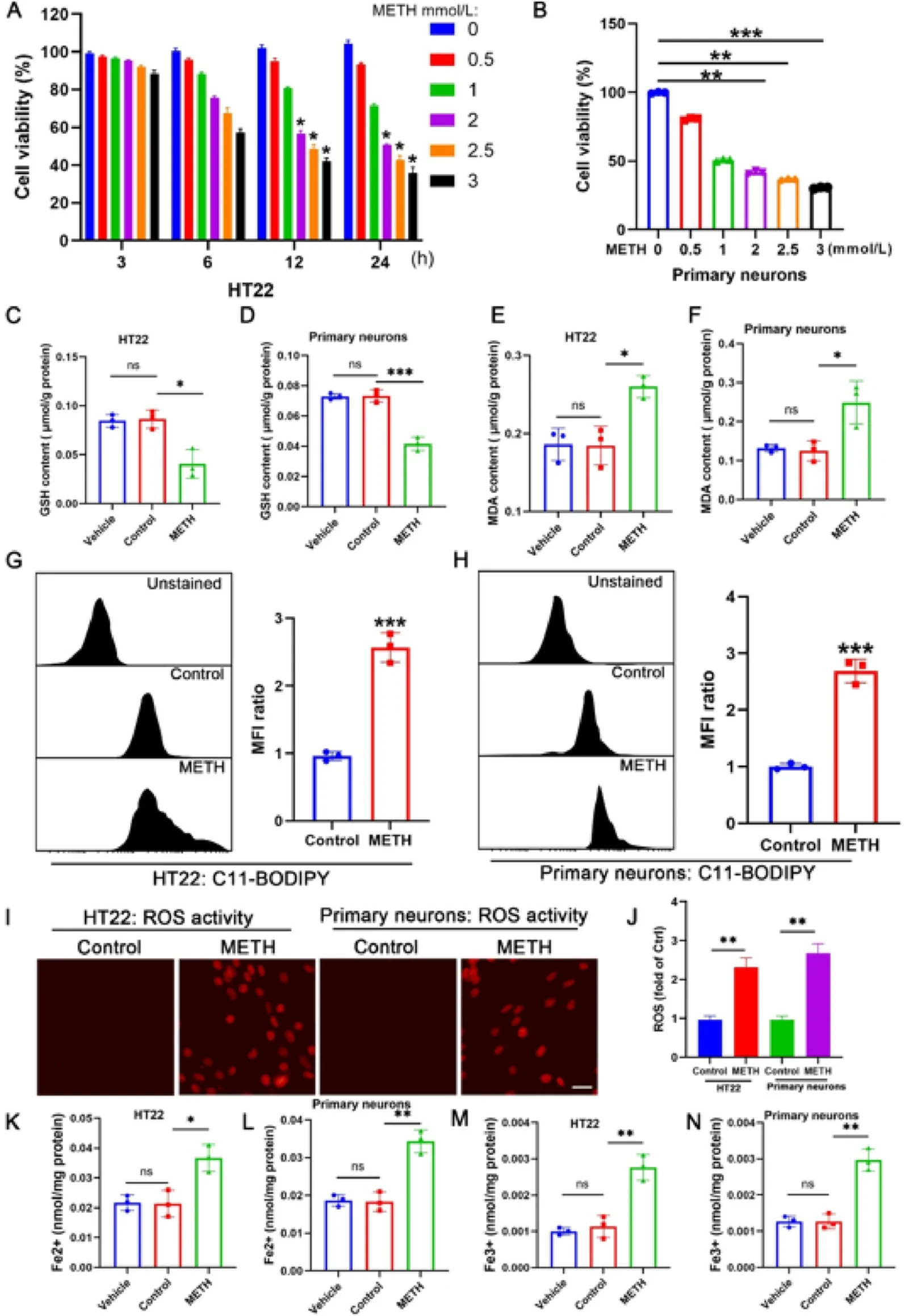
METH-induced ferroptosis in hippocampal neurons. **A** MTS analysis of HT22 cells treated with 0, 0.5, 1.0, 2.0, 2.5, and 3.0 mmol/L METH for 0, 3, 6, 12, and 24 h. **B** MTS analysis of hippocampal-derived primary neurons treated with 0, 0.5, 1.0, 2.0, 2.5, and 3.0 mmol/L METH for 24 h. GSH levels in **C** HT22 cells or **D** hippocampal-derived primary neurons following METH (2 or 1 mmol/L) treatment for 24 h were determined. MDA content in **E** HT22 cells or **F** hippocampal-derived primary neurons following METH (2 or 1 mmol/L) treatment for 24 h was determined. Representative FACS data for C11-BODIPY labeling of **G** HT22 cells or **H** hippocampal-derived primary neurons following METH (2 or 1 mmol/L) treatment for 24 h. Total ROS activity levels were determined in **I** and **J** HT22 cells or hippocampal-derived primary neurons following METH (2 or 1 mmol/L) treatment for 24 h. Intracellular chelatable iron levels (Fe^2+^ and Fe^3+^ levels) were determined using the iron assay kit in **K**–**N** HT22 cells or hippocampal-derived primary neurons following METH (2 or 1 mmol/L) treatment for 24 h. For all figures: Values mean ± SD. One-way ANOVA or two‐way ANOVA. All data are representative of at least three times. *P < 0.05, **P < 0.01, and ***P < 0.001

The hippocampus is a brain region characterized by high metabolic demand and vulnerability to redox stress [26, 27]. To establish the susceptibility of hippocampal neurons to ferroptosis, primary hippocampal neurons were treated with the GPX4 inhibitor RSL3. RSL3 induced a concentration-dependent loss of cell viability, accompanied by a significant increase in MDA levels. The increased MDA levels caused by RSL3 were rescued by ferroptosis inhibitor Fer-1 (Fig. S2A, B), confirming that our neuronal model is sensitive to ferroptosis. Parallel experiments in HT-22 cells using the canonical inducer erastin recapitulated key features of ferroptosis, including the decreased levels of GSH and downregulated GPX4 protein levels (Fig. S2C-F). This profile established hippocampal neurons as susceptible to glutathione-governed ferroptotic cell death, providing a permissive cellular environment for METH-induced damage. To establish a ferroptosis-responsive model, we performed a systematic titration of METH. It elicited a concentration- and time-dependent lethal response in HT-22 cells (Fig. 2A) and, notably, an even greater sensitivity in primary hippocampal neurons (Fig. 2B). Morphological examination of HT-22 cells confirmed hallmarks of ferroptotic death, including cell shrinkage and membrane rupture upon METH exposure (Fig. S3A). Quantitative viability assays (Fig. S3B) guided the selection of optimized METH concentrations (2 mM for HT-22 cells; 1 mM for primary neurons) that provide robust ferroptotic phenotypes while maintaining experimental stability.

**Fig. 3:**
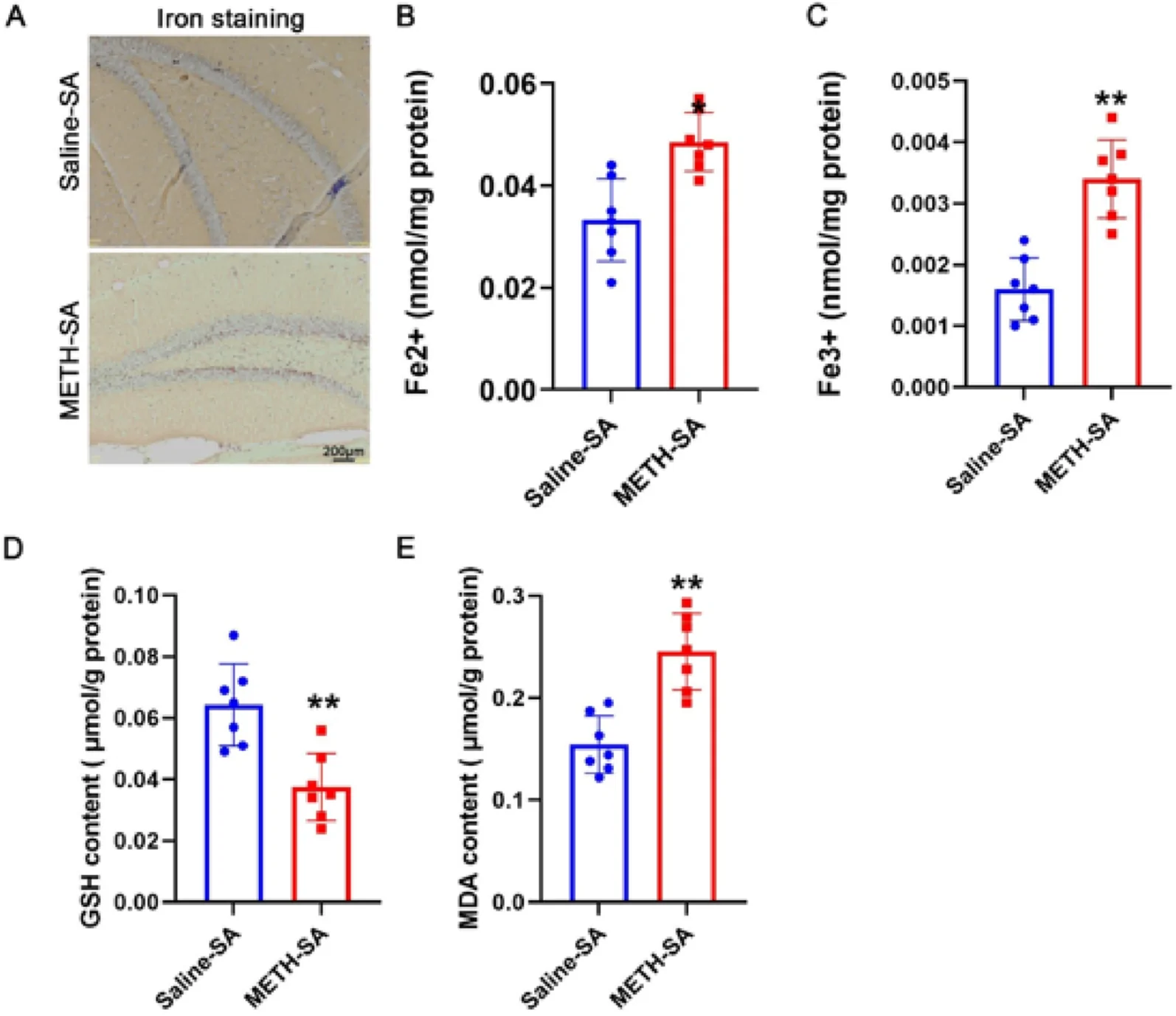
METH-induced ferroptosis in the hippocampus of METH SA mice. **A** DAB-enhanced Perl’s Prussian blue iron staining in the hippocampus of METH SA mice and saline-SA mice at 3 months old. **B**, **C** An iron assay kit was used to measure the Fe^2+^ and Fe^3+^ levels in the hippocampus of METH SA and saline-SA mice at 3 months old. **D** GSH levels and **E** MDA levels were determined in the hippocampus of METH SA mice and saline-SA mice at 3 months old. For all figures: Values mean ± SD. *N* = 8 mice/group. One-way ANOVA or two‐way ANOVA. *P < 0.05, **P < 0.01, and ***P < 0.001. All data are representative of at least three times

Mechanistic characterization of cell death revealed a canonical ferroptotic signature. METH exposure triggered a collapse of GSH homeostasis (Fig. 2C, D) and a concomitant accumulation of MDA, a marker of lipid peroxidation (Fig. 2E, F). Real-time monitoring using the C11-BODIPY probe confirmed a marked acceleration in lipid peroxidation rates (Fig. 2G-J). Furthermore, METH induced a significant expansion of the labile iron pool (Fig. 2K-N, Fig. S3E). Cell death under these conditions was determined to be caspase-independent (Fig. S3C), consistent with ferroptosis rather than apoptosis. A crucial temporal analysis revealed that GSH depletion is an early, precipitating event in this cascade. A significant drop in intracellular GSH was detectable as early as 3 h post-METH treatment (Fig. S3D), a time point when cell viability remained high (Fig. 2A) and lipid peroxidation was only beginning. The substantial loss of viability occurred at later time points (18–24 h), placing GSH depletion upstream of the execution phase. Finally, we validated this ferroptotic cascade in vivo. In the hippocampi of mice following chronic METH SA, we observed a neuropathological triad characteristic of ferroptosis: significant iron overload, profound GSH depletion, and elevated MDA accumulation (Fig. 3A-E). Collectively, these data from cellular models and in vivo validation demonstrate an intrinsic vulnerability of hippocampal neurons to a METH-driven ferroptotic cascade.

### Ferroptosis inhibition protects against METH-induced neuronal death and cognitive impairment

**Fig. 4:**
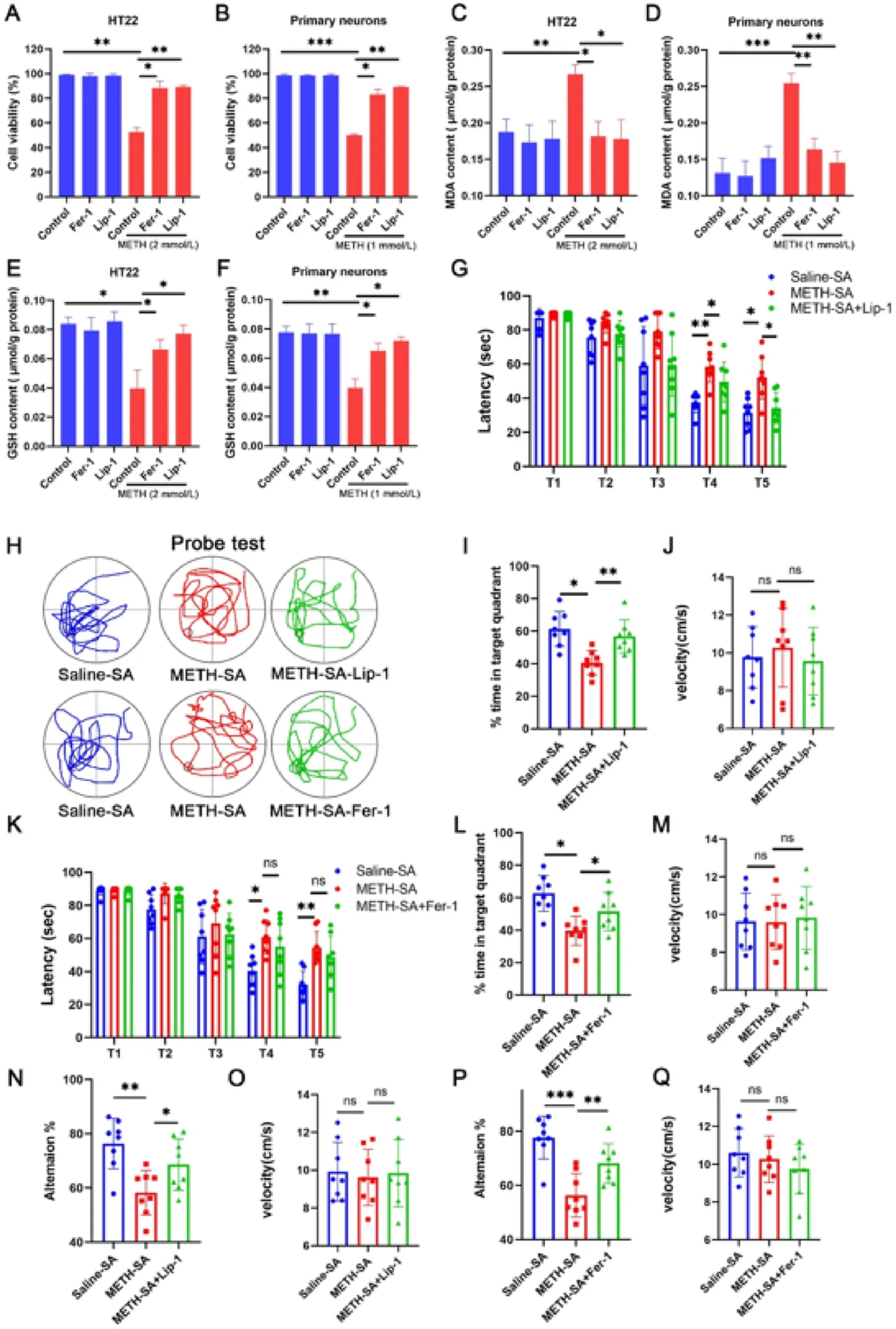
Inhibition of ferroptosis prevents METH-induced hippocampal neuron death and cognitive decline. MTS analysis of cell viability in **A** HT22 cells or **B** hippocampal-derived primary neurons following METH (2 or 1 mmol/L) treatment with either Fer-1 (1 µM) or Lip-1 (100 nM) for 24 h. MDA contents in **C** HT22 cells or **D** hippocampal-derived primary neurons following METH (2 or 1 mmol/L) treatment with either Fer-1 (1 µM) or Lip-1 (100 nM) for 24 h were measured. SH levels in **E** HT22 cells or **F** hippocampal-derived primary neurons following METH (2 or 1 mmol/L) treatment with either Fer-1 (1 µM) or Lip-1 (100 nM) for 24 h were measured. **G** Escape latencies (s) of saline-SA, METH SA, and METH SA + Lip-1 mice in the MWM test. **H** Swim paths, **I** percentage of time spent swimming in the target quadrant, and **J** average velocity during the probe test for mice in the MWM test. **K** Escape latencies (s) of saline-SA, METH SA, and METH SA + Fer-1 mice in the MWM test. **H** Swim paths, **L** percentage of time spent swimming in the target quadrant, and **M** average velocity during the probe test for mice in the MWM test. **N** and **P** Alternation of arms in the spontaneous alternation test of mice in the Y-maze test and **O** and **Q** average velocity during the spontaneous alternation test of mice in the Y-maze test. For all figures: Values mean ± SD. *N* = 8 mice/group. One-way ANOVA or two‐way ANOVA. *P < 0.05, **P < 0.01, and ***P < 0.001. All data are representative of at least three times

To establish a causal role for ferroptosis in METH-induced neurotoxicity, we employed two mechanistically distinct ferroptosis inhibitors: Lip-1 [28] (100 nM), which suppresses lipid peroxidation, and Fer-1 [29] (1 µM), a radical-trapping antioxidant. Both inhibitors conferred robust protection, significantly rescuing cell viability in METH-treated HT-22 cells and primary hippocampal neurons (Fig. 4A, B). Biochemical analysis confirmed that the protective effects were mediated through ferroptosis-specific pathways. Co-treatment with either inhibitor markedly attenuated METH-induced lipid peroxidation, as shown by a significant reduction in MDA levels (Fig. 4C, D). Concurrently, the depletion of GSH was effectively prevented, with cellular GSH levels maintained near baseline (Fig. 4E, F).

We further assessed the functional relevance of ferroptosis inhibition in vivo using our METH SA model. An independent cohort of mice received either Lip-1 or Fer-1 during behavioral testing. Both inhibitors ameliorated METH-induced cognitive deficits, but with differential efficacy. In the MWM, Lip-1 treatment fully restored spatial memory, significantly improving escape latency during training and increasing target quadrant preference during the probe trial (Fig. 4G-J). Fer-1 provided partial protection, showing significant improvement in probe trial performance but a more modest effect on escape latency during training (Fig. 4K-M). In the Y-maze test, both Lip-1 and Fer-1 significantly rescued spontaneous alternation performance impaired by METH exposure (Fig. 4N-Q). These results demonstrate that pharmacological inhibition of ferroptosis is sufficient to prevent METH-induced hippocampal neuronal death and cognitive decline, providing direct causal evidence that ferroptosis is a key driver of METH-related neuropathology.

### METH-induced ferroptosis is driven by GPX4 proteolytic degradation

Initial investigation revealed that although METH exposure induces significant ROS accumulation, scavenging these ROS with NAC failed to prevent the associated lipid peroxidation or GSH depletion, indicating that ferroptosis initiation in this context is ROS-independent (Fig. S4A-H).

**Fig. 5:**
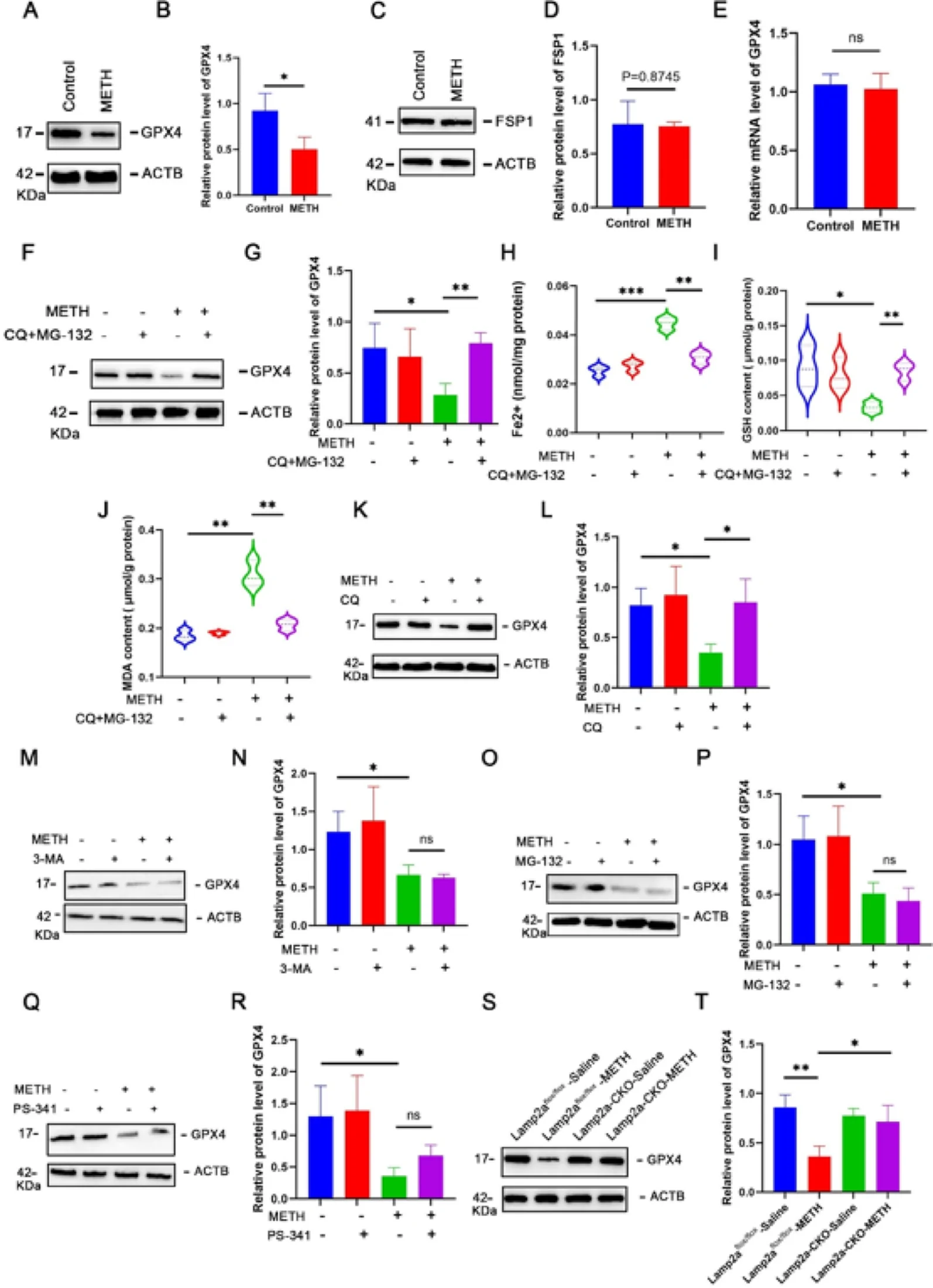
GPX4 degradation plays a key role in METH-induced ferroptosis. Western blotting analysis was used to detect the protein level of **A** GPX4 or **C** FSP1 in HT22 cells treated with erastin (0, 1, 2.5, or 5 µM) for 24 h. **B** Relative GPX4 protein level. **D** Relative FSP1 protein level. **E** qRT-PCR analysis was used to detect the mRNA level of GPX4. HT-22 cells were pretreated with 25 µM CQ + 10 µM MG-132, 25 µM CQ, 25 µM 3-MA, 10 µM MG-132, or 500 nM PS-341 for 1 h and treated with METH for 12 h. **F**, **K**, **M**, **O**, and **Q** Western blotting analysis was used to detect the protein levels of GPX4 after different treatments. **G**, **L**, **N**, **P**, and **R** Relative GPX4 protein levels. Fe2 + levels **H**, GSH levels **I** and MDA contents **J** in HT22 cells following CQ + MG-132 treatment for 1 h and treated with METH for 12 h were determined. Western blotting analysis was used to detect the protein level of **S**GPX4 in primary hippocampal neurons from neuronal-specific CMA-deficient mice. **T** Relative GPX4 protein level. For all figures: Values mean ± SD. One-way ANOVA or two‐way ANOVA. All data are representative of at least three times. *P < 0.05, **P < 0.01, and ***P < 0.001

We therefore systematically examined key ferroptosis surveillance systems. We found that METH exposure led to a significant depletion of the core ferroptosis inhibitor GPX4 at the protein level, without affecting its mRNA expression (Fig. 5A-E). In contrast, the protein stability of the backup regulator FSP1 remained unchanged (Fig. 5C, D).

To first confirm that GPX4 loss was mediated by protein degradation, we co-treated cells with a combination of lysosomal and proteasomal inhibitors (CQ + MG-132). This combined inhibition fully rescued METH-induced lipid peroxidation (Fig. 5F-J). Subsequently, to dissect the responsible pathway, individual application of either CQ or MG-132 also showed significant protective effects against these downstream phenotypes (Fig. 5K-R). Finally, to establish the specific lysosomal pathway involved, we employed a genetic model. In Lamp2a-CKO mice, the degradation of GPX4 following METH exposure was significantly attenuated compared to control mice (Fig. 5S, T). These data demonstrate that METH triggers ferroptosis primarily through the lysosomal degradation of GPX4, a process that is independent of transcriptional downregulation and general ROS accumulation.

**Fig. 6:**
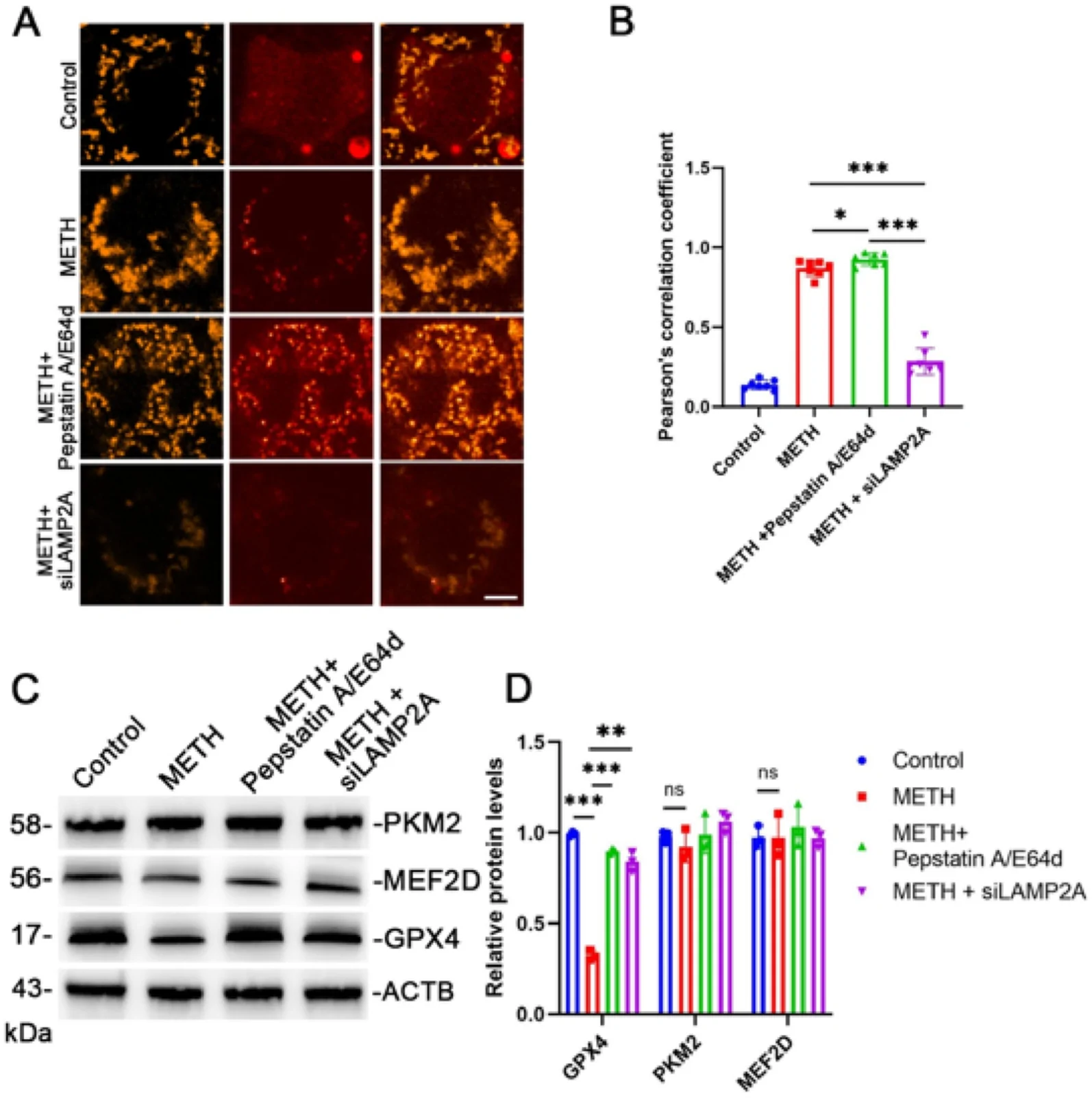
METH induces GPX4 translocation into lysosomes via a CMA-dependent mechanism. **A** Representative confocal microscopy images of HT22 cells stained for GPX4 (red), and the lysosomal marker LAMP2A (yellow). Scale bar: 10 μm. Control: Basal level of co-localization, METH + Pepstatin A/E64d: Dramatic accumulation of GPX4 within LAMP2A-positive lysosomes. METH + siLAMP2A: Knockdown of the CMA receptor LAMP2A prevents METH-induced GPX4 lysosomal targeting. **B** Quantification of the Pearson’s correlation coefficient between GPX4 and LAMP2A signals. Data are mean ± SD; *n* = 7, *p < 0.001 vs. Control. **C** Representative western blotting showing PKM2, MEF2D and GPX4 proteins. **E** Bar graph quantifying proteins relative levels from western blotting (*n* = 3 independent experiments). Data are mean ± SD; Not significant. *p < 0.001 vs. Control

To conclusively demonstrate that GPX4 degradation occurs through CMA translocation into lysosomes, we blocked the final intra-lysosomal proteolysis step using a combination of pepstatin A (aspartyl protease inhibitor) and E64d (cysteine protease inhibitor). Compared to control cells, METH treatment alone significantly increased the co-localization of GPX4 with LAMP2A-positive lysosomes. Crucially, co-treatment with METH and these inhibitors caused a dramatic accumulation of GPX4 signal within the lysosomal lumen (Fig. 6A, B). This pattern is diagnostic of CMA inhibition: the substrate is successfully translocated into lysosomes but accumulates due to arrested degradation, providing direct visualization that GPX4 is actively transported into the lysosomal interior, not merely associated with the membrane. This CMA-specificity was confirmed genetically, as siRNA-mediated knockdown of LAMP2A completely abolished METH-induced GPX4-lysosome co-localization (Fig. 6A, B).

To determine if METH generally enhances CMA activity, we analyzed two canonical CMA substrates, MEF2D and PKM2, alongside GPX4. While METH exposure specifically reduced GPX4 protein levels, it did not alter MEF2D or PKM2 abundance (Fig. 6C, D). Furthermore, reducing GPX4 degradation via pharmacological (pepstatin A/E64d) or genetic (siLAMP2A) CMA inhibition had no effect on MEF2D or PKM2 stability. This demonstrates that METH selectively engages CMA to target GPX4 rather than causing a generalized hyperactivation of the pathway.

These findings provide direct visual and functional evidence that GPX4 is selectively trafficked via the CMA machinery into lysosomes for degradation, and that this specific event is a central driver of the ensuing ferroptotic cascade induced by METH.

### METH promotes CMA-dependent GPX4 degradation via HSC70-mediated substrate recognition

**Fig. 7:**
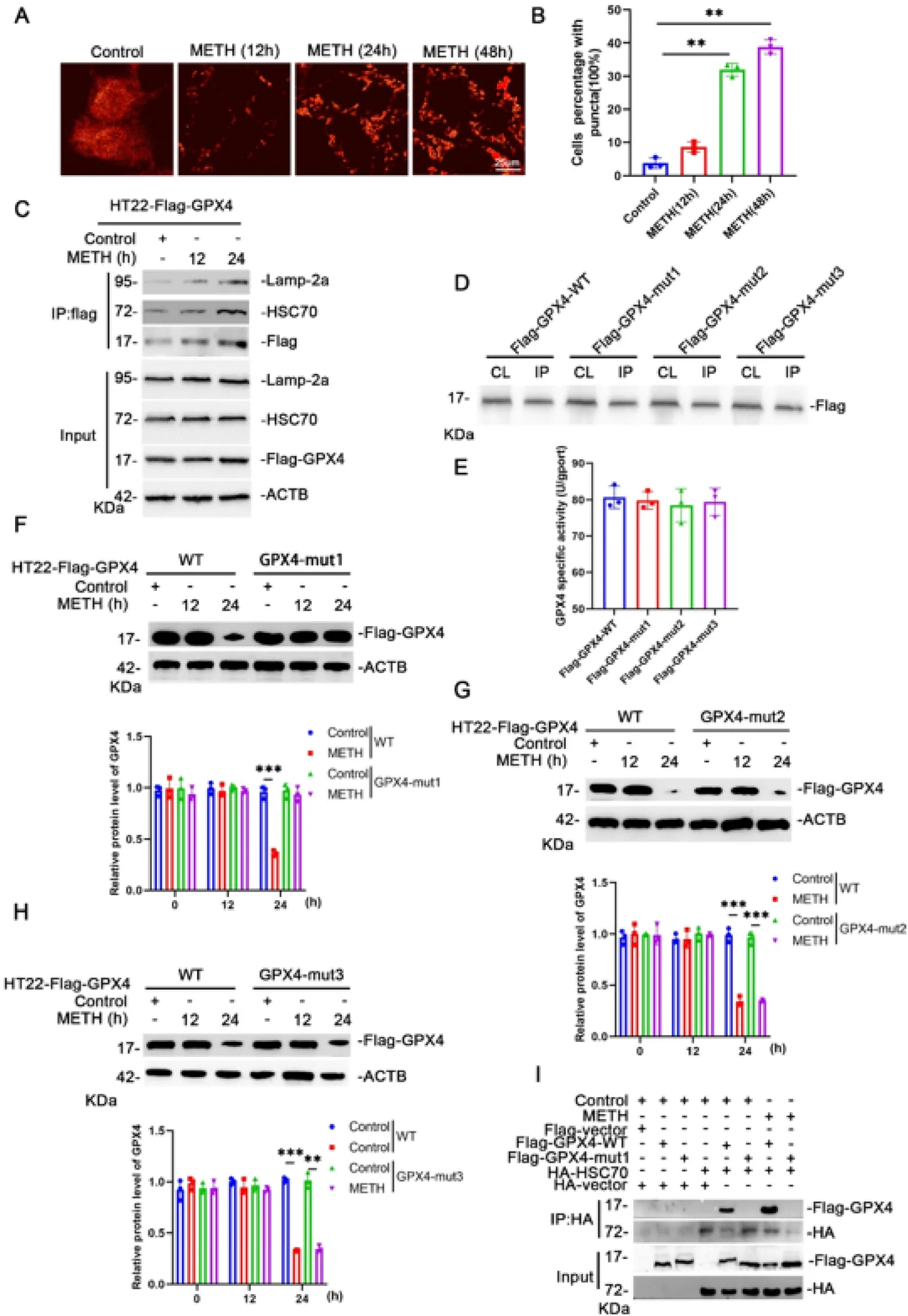
GPX4 degradation that occurs in lysosomes is mediated by CMA upon METH exposure. **A** HT22 cells stably expressed KFERQ-PA-mCherry were activated in a 405 nm wave and exposed to METH for 12, 24, or 48 h. **B** ImageJ software was used for quantification. **C** HT22-Flag-GPX4 cells were treated with METH for 12–24 h. The cell lysates were subjected to IP using an anti-flag antibody and then analyzed by Western blotting. **D** Representative immunoblot showing FLAG-immunoprecipitated WT and mutant GPX4 proteins used for the activity assay. **E** Bar graph quantifying GPX4 enzymatic activity from immunoprecipitates (*n* = 3 independent experiments). Activity is normalized to the amount of immunoprecipitated GPX4 protein and expressed relative to WT GPX4. Data are mean ± SD; Not significant. **F**–**H** HT22-Flag-GPX4 cells or HT22 cells stably expressed the indicated Flag-GPX4 mutants were treated with METH for 12–24 h. The cell lysates were subjected to Western blotting using the indicated antibodies. **I** Flag-tagged GPX4, GPX4-mut1, and HA-tagged HSC70 expression plasmids were co-transfected to HEK293T cells for 24 h. The cell lysates were subjected to IP with an anti-HA antibody and then analyzed by Western blotting. For all figures: Values mean ± SD. One-way ANOVA or two‐way ANOVA. All data are representative of at least three times. *P < 0.05, **P < 0.01, and ***P < 0.001

To assess CMA activation, we employed a photoactivatable KFERQ-PA-mCherry reporter, observing a significant increase in lysosomal translocation following METH exposure (2 mM, 48 h), indicative of enhanced CMA flux (Fig. 7A, B). We then established an HT22 cell line stably expressing FLAG-tagged GPX4 to enable co-immunoprecipitation assays. After METH treatment, we detected markedly strengthened interactions among HSC70, LAMP2A, and GPX4, suggesting enhanced recognition and targeting of GPX4 by the CMA machinery (Fig. 7C).

Bioinformatic analysis identified three putative CMA-targeting motifs within GPX4: ^169^LIDKN^173^, ^124^NVKFD^125^, and ^187^QVIEK^191^. To investigate their functional relevance, we generated GPX4 mutants with double-alanine substitutions in each motif (GPX4-mut1, mut2, mut3). Importantly, in vitro enzyme activity assays confirmed that these mutations did not impair GPX4 catalytic function, as all mutants retained enzymatic activity comparable to wild-type GPX4 (Fig. 7D, E). We next examined the degradation kinetics of these mutants under METH challenge. Notably, only GPX4-mut1 (carrying mutations in the ^124^NVKFD^125^ motif) exhibited significant resistance to METH-induced degradation (Fig. 7F–H). Consistent with this, co-immunoprecipitation experiments revealed a loss of interaction between GPX4-mut1 and HSC70 (Fig. 7I).

These results demonstrate that METH triggers GPX4 degradation via the CMA pathway through specific recognition of a targeting motif (^124^NVKFD^125^) by HSC70, thereby establishing a direct mechanistic link between CMA activation and ferroptosis execution.

### Inhibition of CMA reverses METH-induced neurobehavioral deficits by attenuating ferroptosis

**Fig. 8:**
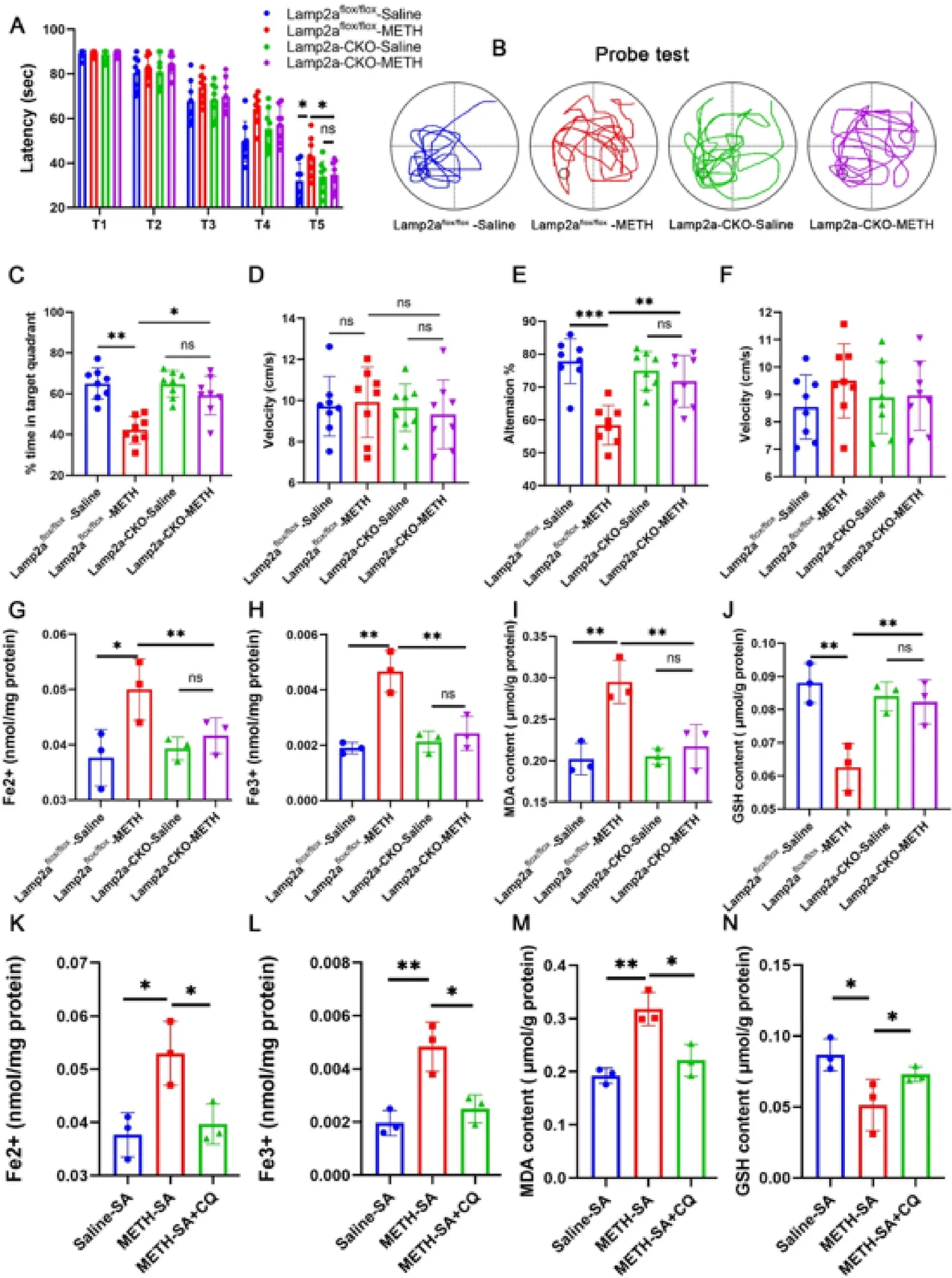
Inhibition of CMA ameliorated METH-induced ferroptosis and cognitive decline. **A** Escape latencies (s) of Lamp2a^flox/flox^-saline, Lamp2a^flox/flox^-METH, Lamp2a-CKO-saline, and Lamp2a-CKO-METH mice in the MWM test. **B** Swim paths, **C** percentage of time spent swimming in the target quadrant, and **D** average velocity during the probe test for mice in the MWM test. **E** Alternation of arms in the spontaneous alternation test of mice in the Y-maze test and **F** average velocity during the spontaneous alternation test of mice in the Y-maze test. **G**, **H** An iron assay kit was used to measure Fe^2+^ and Fe^3+^ levels in the hippocampus of Lamp2a^flox/flox^-saline, Lamp2a^flox/flox^-METH, Lamp2a-CKO-saline, and Lamp2a-CKO-METH mice at 3 months old. **I** MDA contents in the hippocampus of Lamp2a^flox/flox^-saline, Lamp2a^flox/flox^-METH, Lamp2a-CKO-saline, and Lamp2a-CKO-METH mice at 3 months old were measured. **J** GSH levels in the hippocampus of Lamp2a^flox/flox^-saline, Lamp2a^flox/flox^-METH, Lamp2a-CKO-saline, and Lamp2a-CKO-METH mice at 3 months old were measured. **K**−**L** An iron assay kit was used to measure the Fe^2+^ and Fe^3+^ levels in the hippocampus of saline-SA, METH SA, and METH SA + CQ mice at 3 months old. (**M**MDA contents in the hippocampus of saline-SA, METH SA, and METH SA + CQ mice at 3 months old were measured. **N** GSH levels in the hippocampus of saline-SA, METH SA, and METH SA + CQ mice at 3 months old were measured. For all figures: Values mean ± SD. One-way ANOVA or two‐way ANOVA. *N* = 8 mice/group. *P < 0.05, **P < 0.01, and ***P < 0.001. All data are representative of at least three times

Comprehensive behavioral phenotyping of mice with conditional knockout of Lamp2a in hippocampal neurons revealed multi-domain therapeutic benefits consequent to CMA blockade. First, genetic inhibition of CMA rescued METH-induced cognitive deficits. In the MWM, Lamp2a-CKO mice exhibited a significantly shorter escape latency during training and a pronounced increase in target quadrant occupancy during the probe trial, compared to Lamp2a^flox/flox^ control mice (Fig. 8A-D). Furthermore, spontaneous alternation performance in the Y-maze was significantly improved in Lamp2a-CKO mice (Fig. 8E-F). Second, CMA blockade attenuated METH-seeking behaviors. Under a FR1 reinforcement schedule, Lamp2a-CKO mice displayed a significant reduction in both active nose-poke responses and total METH infusions (Fig. S5A-B). This effect persisted under a more demanding progressive-ratio schedule, where Lamp2a-CKO mice achieved significantly lower breakpoints (Fig. S5C). At the molecular level, hippocampal iron overload and neurons death induced by METH was markedly attenuated in Lamp2a-CKO mice (Fig. 8G-H, Fig. S7). Consistent with a reduction in ferroptotic stress, lipid peroxidationwas decreased (Fig. 8I), while the antioxidant GSH was significantly increased in the hippocampus of Lamp2a-CKO mice following METH exposure (Fig. 8J).

Complementary pharmacological studies using the lysosomal/CMA inhibitor CQ corroborated these findings. In vitro, CQ pretreatment protected HT22 cells and primary hippocampal neurons from METH-induced cell death, iron accumulation, and lipid peroxidation (Fig. S6A-H). To validate the specificity of CMA inhibition in blocking ferroptosis, HT22 cells were treated with erastin. Erastin triggered ferroptosis, elevating the labile iron pool and MDA while depleting GSH. Co-treatment with the CMA inhibitor CQ effectively countered these changes (Fig. S6I-L), confirming its role as a ferroptosis inhibitor and linking CMA blockade to suppression of this death pathway. In vivo, administering CQ to mice prior to and during METH SA significantly ameliorated cognitive deficits in the MWM and Y-maze (Fig. S6M-R). Furthermore, CQ treatment reduced METH-seeking behavior under both FR1 and PR schedules (Fig. S5D-F) and attenuated the associated hippocampal ferroptotic markers—iron overload, elevated MDA, and GSH depletion—in METH SA mice (Fig. 8K-N). In summary, our data demonstrate that METH addiction activates CMA, leading to GPX4 degradation, ferroptosis, and subsequent cognitive and behavioral deficits across multiple translational models. Genetic inhibition of neuronal CMA was sufficient to prevent these pathological and behavioral outcomes.

## Discussion

Our study proposes a novel mechanism for METH-induced neurotoxicity, in which the drug hijacks the CMA pathway to degrade the antioxidant enzyme GPX4, thereby triggering ferroptosis and contributing to the cognitive deficits associated with addiction. This model is supported by evidence from: (1) METH promotes CMA-dependent degradation of GPX4 via a putative motif resembling a CMA-targeting sequence; (2) Loss of GPX4 impairs antioxidant capacity, leading to lipid peroxidation and neuronal damage; (3) Inhibition of CMA in neurons ameliorates spatial memory deficits and reduces drug-seeking behavior, suggesting a role for CMA activity in addiction-related pathophysiology. Collectively, these findings provide new insights into how dysregulation of proteostasis contributes to neuronal damage and suggest that the CMA-GPX4 axis may represent a promising therapeutic target for mitigating cognitive decline in stimulant use disorders.

The CMA pathway is widely recognized for its indispensable role in maintaining neuronal proteostasis and survival under physiological conditions. By selectively degrading soluble proteins bearing a KFERQ-like motif, CMA facilitates the continuous removal of damaged or misfolded proteins, thereby preventing toxic aggregate accumulation, a process particularly crucial in post-mitotic neurons [30]. This constitutive quality-control function is generally regarded as cytoprotective, and its decline or dysfunction has been implicated in the pathogenesis of various neurodegenerative disorders. For instance, CMA activity is compromised in models of Alzheimer’s [31] and Parkinson’s [32] diseases, contributing to the accumulation of pathogenic proteins like α-synuclein and Tau, whereas its enhancement can ameliorate associated pathologies. Thus, within a homeostatic or slowly progressing pathological context, CMA operates primarily as an adaptive, pro-survival mechanism.

In striking contrast, our findings reveal that under the specific, intense proteotoxic stress imposed by chronic METH exposure, CMA can be pathologically subverted. We demonstrate that METH triggers a pronounced upregulation of CMA activity components (LAMP2A, HSC70). However, rather than executing a broad protective clearance, this activated CMA appears to be preferentially directed towards the degradation of GPX4, a pivotal antioxidant enzyme containing a putative KFERQ-like motif. This specific substrate targeting converts a normally protective proteolytic system into a direct mediator of ferroptotic cascades. The resultant depletion of GPX4 undermines cellular defense against lipid peroxidation, leading to irreversible neuronal damage. This delineates a clear condition-dependent functional duality of CMA: its outcome is contingent upon the cellular environment and the specific substrates engaged. Under basal or mild stress, its activity is adaptive and vital for health; under the acute, sustained insult of a potent psychostimulant like METH, its over-activation and/or substrate selectivity shift becomes maladaptive, actively driving neurodegeneration. Our work thereby positions CMA not merely as a passive responder but as a dynamic, context-dependent switch whose role transitions from guardian to executioner in the face of severe metabolic and oxidative challenge.

Our work established that METH-induced ferroptosis is initiated independently of the general ROS surge, as scavenging ROS with NAC failed to prevent cell death. This guided us to discover the central role of CMA-dependent GPX4 degradation. The mechanism of GSH depletion is not a simple cause but a catastrophic consequence of the ensuing lipid peroxidation, creating a self-amplifying vicious cycle: METH-induced CMA activation leads to the degradation of GPX4, the key enzyme responsible for reducing phospholipid hydroperoxides. Unchecked lipid peroxidation generates a cascade of toxic lipid-derived reactive species. These lipid peroxidation products directly inhibit system Xc- activity, as evidenced by our new data showing METH-induced downregulation of SLC7A11, the functional subunit of system Xc- (Supplementary Fig. S4I, J). Crucially, this downregulation is ameliorated by CMA inhibition, mechanistically linking it to our core pathway. Inhibition of system Xc- impairs cystine uptake, starving the cell of the rate-limiting precursor for *de novo* GSH synthesis. This failure in GSH synthesis, coupled with the continued consumption of GSH by other antioxidant pathways overwhelmed by METH-induced stress, leads to irreversible depletion of the GSH pool. Thus, GPX4 loss is not the direct cause of GSH depletion but the critical trigger that dismantles the primary defense against lipid peroxidation. The resulting lipid-derived toxins then disrupt the very system (Xc-) needed to replenish GSH, creating a feed-forward loop that seals the cell’s fate. This model coherently explains why NAC (which targets ROS but not lipid peroxides or system Xc- function) was ineffective, and positions GPX4 loss as the pivotal event that converts a stressed state into a catastrophic metabolic collapse.

The increase in LIP is a recognized hallmark and amplifier of ferroptosis. Our data suggest that METH-induced LIP expansion is mediated through the activation of ferritinophagy, the selective autophagic degradation of the iron-storage protein ferritin. We observed that METH treatment significantly upregulates the expression of NCOA4, the key cargo receptor for ferritinophagy^33^, in hippocampal neurons (Fig. S4I, J). Importantly, pharmacological inhibition of CMA attenuated METH-induced NCOA4 upregulation and subsequent LIP increase. This places ferritinophagy activation downstream of the hijacked CMA pathway. In conclusion, we propose that METH-induced CMA activation orchestrates ferroptosis by simultaneously disabling the primary antioxidant defense and enhancing the pro-ferroptotic milieu. The GSH depletion is a consequence of the system’s collapse under sustained oxidative insult after the loss of its key guardian, GPX4.

Based on our findings, we propose that CMA hyperactivation acts as an early precipitating factor in METH-induced neurodegeneration, rather than a late-stage epiphenomenon of chronic toxicity. This conclusion is supported by a clear temporal sequence: CMA activation and subsequent GPX4 degradation precede substantial neuronal loss and cognitive decline. The functional necessity of this pathway is underscored by rescue experiments, where CMA inhibition, even after chronic METH exposure, effectively attenuated ferroptosis and restored cognitive function. Furthermore, the specific targeting of GPX4, but not other CMA substrates, indicates a programmed hijacking of a selective proteolytic system. Thus, CMA hyperactivation serves as both the initial trigger and a sustaining driver of the pathological cascade, positioning it as a compelling early therapeutic target for intervention.

A critical consideration in this study is the inherent limitation of the pharmacological tools employed to modulate autophagy. Compounds such as CQ and 3-MA, while useful for initial exploration, are broad-spectrum inhibitors of lysosomal acidification and early-stage autophagy, respectively. They lack specificity for the CMA pathway and may confer off-target effects that complicate data interpretation. To decisively overcome this fundamental limitation and establish a causal link specifically to CMA, our experimental design critically incorporated a genetic approach. The generation and analysis of Lamp2a-CKO mice provided a direct, loss-of-function model to assess the consequences of neuron-specific CMA ablation. The concordance between the protective phenotypes observed in Lamp2a-CKO mice and the outcomes following pharmacological inhibition strongly reinforces the conclusion that the observed effects are mediated specifically by CMA activity, rather than by non-specific drug actions. This complementary use of pharmacological and genetic strategies significantly strengthens the methodological rigor and validity of our central claim.

Several other limitations provide avenues for future research. While our focus on hippocampal neurons is justified by the central role of this region in the cognitive deficits of addiction, it will be important to determine whether the described CMA-GPX4 axis operates in other brain regions implicated in reward and compulsion. Furthermore, although our genetic model strongly supports a causal role for CMA, the precise molecular mechanism by which METH exposure directs CMA machinery specifically towards GPX4 degradation warrants further elucidation. Future studies employing techniques such as substrate-specific CMA flux assays or proteomic analysis of CMA-targeted proteins under METH exposure will help refine this mechanistic understanding.

In this study, ferroptosis and CMA inhibitors were administered via the intranasal (i.n.) route. This approach was chosen based on its well-established capacity to facilitate direct drug delivery to the central nervous system by bypassing the blood-brain barrier, offering a non-invasive strategy to maximize target engagement in the brain for neuroprotective agents [34]. While our functional data robustly demonstrate the efficacy of this route in mitigating METH-induced pathology, detailed pharmacokinetic profiles of Lip-1, Fer-1, and CQ following i.n. administration remain to be fully characterized. This represents a point for future investigation, which would further optimize dosing paradigms for therapeutic translation.

Our findings extend the current understanding of METH-induced neurotoxicity by moving beyond the established paradigm of generalized oxidative stress. While prior research has consistently documented elevated reactive oxygen species and lipid peroxidation following METH exposure [35], our work identifies a critical upstream proteostatic mechanism driving this oxidative insult: the dysregulation of CMA leading to the precise depletion of the master antioxidant regulator, GPX4. This shifts the focus from the downstream consequences of oxidative stress to a manipulable upstream node, offering a more targeted mechanistic framework for intervention.

Furthermore, this study significantly expands the role of autophagy in addiction pathology. The vast majority of previous investigations have centered on the contributions of macroautophagy [36], a bulk degradation process. By contrast, we elucidate a pathophysiological function for CMA, a distinctly selective and mechanistically discrete form of autophagy. This underscores the emerging concept that different autophagic pathways can play divergent, even opposing, roles within the same disease context. Our data suggest that a nuanced understanding of autophagic subtypes is essential for accurately deciphering their contributions to neuropsychiatric disorders.

Our study identifies CMA as a specific degradation route for GPX4 in the context of METH neurotoxicity. Intriguingly, recent landmark studies in multiple sclerosis (MS) have highlighted parallel, yet distinct, mechanisms targeting the same ferroptotic defense axis. In MS, neuronal inflammatory stress activates the STING pathway, which in turn impairs glutathione biosynthesis, thereby sensitizing neurons to ferroptosis [37]. Simultaneously, the immunoproteasome, upregulated in MS, directly targets GPX4 for degradation, establishing a direct link between neuroinflammation and the loss of this key antioxidant enzyme [38].

Together with our findings, a converging picture emerges: the GPX4/GSH axis represents a critical vulnerability node in neurons across diverse CNS disorders. Whether triggered by exogenous toxins via CMA, or by endogenous neuroinflammation via the immunoproteasome or STING-mediated autophagy, the ultimate outcome is a collapse of lipid peroxidation repair, leading to ferroptotic neuronal death. This underscores the potential of ferroptosis inhibition as a universal therapeutic strategy. Furthermore, our work adds CMA to the repertoire of cellular quality-control systems that can be hijacked to deplete GPX4, suggesting that the therapeutic protection of neurons may require precise targeting of the relevant upstream degradation machinery specific to each disease context.

The mechanistic delineation of the CMA-GPX4 axis provides a compelling theoretical foundation for therapeutic targeting. Instead of broadly mitigating oxidative stress or inhibiting autophagy in its entirety, our work posits that the precise modulation of CMA activity, specifically to prevent the maladaptive degradation of GPX4, represents a novel and potentially more effective strategy. This presents a clear trajectory for future drug discovery efforts aimed at developing specific CMA modulators, such as small molecules that disrupt the LAMP2A-GPX4 interaction or enhance GPX4 stability, to mitigate cognitive decline in stimulant use disorders without globally compromising protein homeostasis.

## Supplementary Information

Below is the link to the electronic supplementary material.

Supplementary Material 1

Supplementary Material 2

## Data Availability

No datasets were generated or analyzed during the current study.
