# Supplementary material for: Methamphetamine hijacks chaperone-mediated autophagy to degrade GPX4, driving ferroptosis-precipitated cognitive decline and addictive pathogenesis
Source: Acta Neuropathol Commun. 2026 Jun 15;14:177. doi: 10.1186/s40478-026-02342-7 (PMC13501791; doi:10.1186/s40478-026-02342-7)

Figure 4A

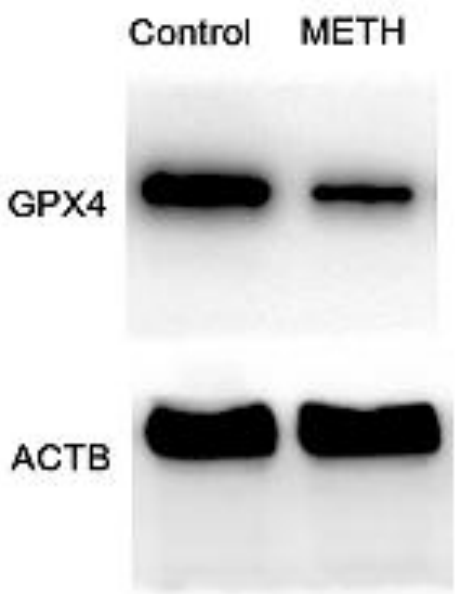


Figure 4C

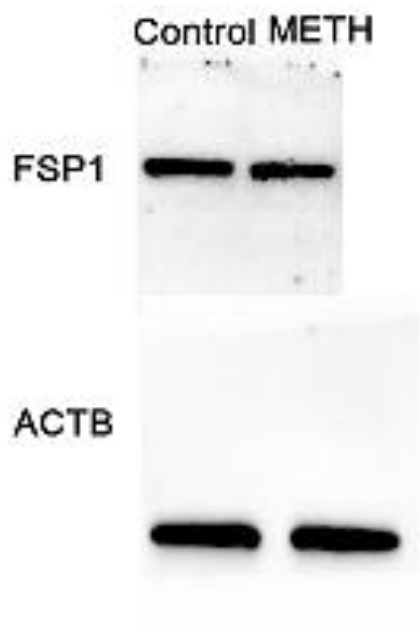


Figure 4F

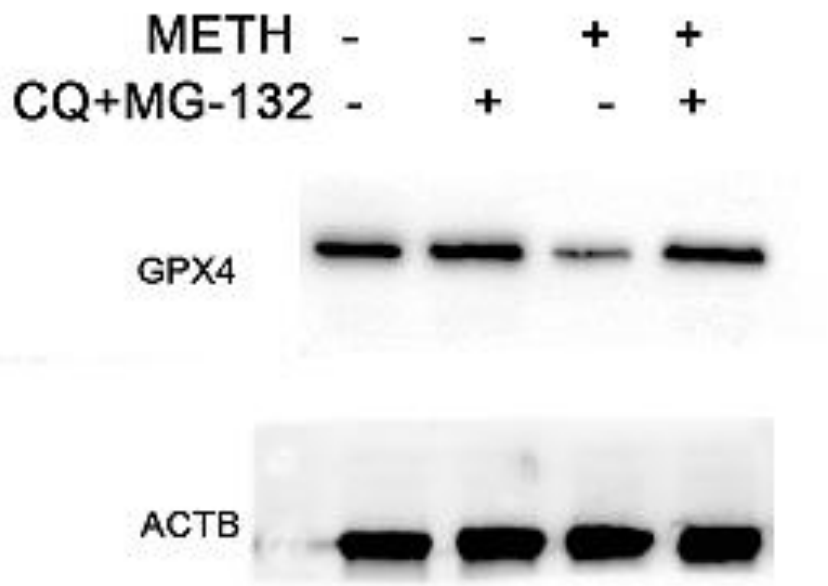


Figure 4K

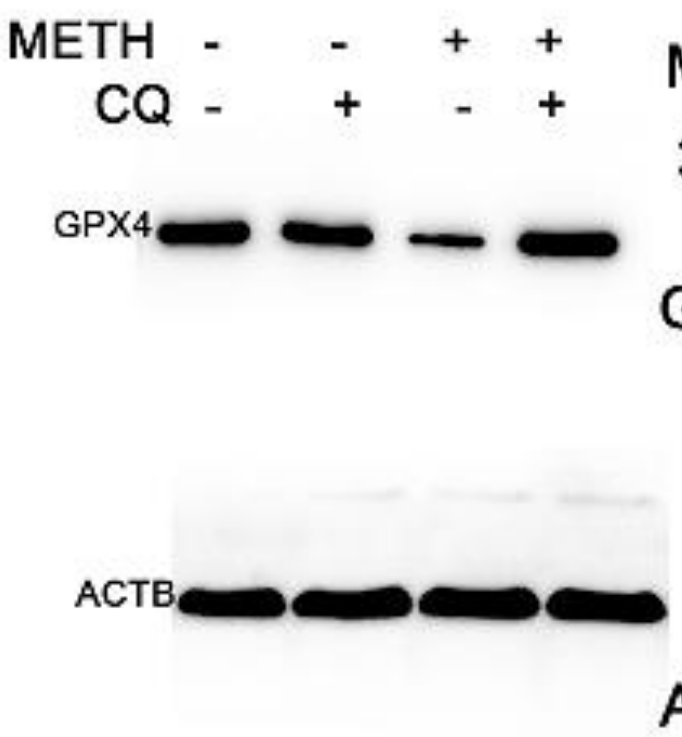


Figure 4M

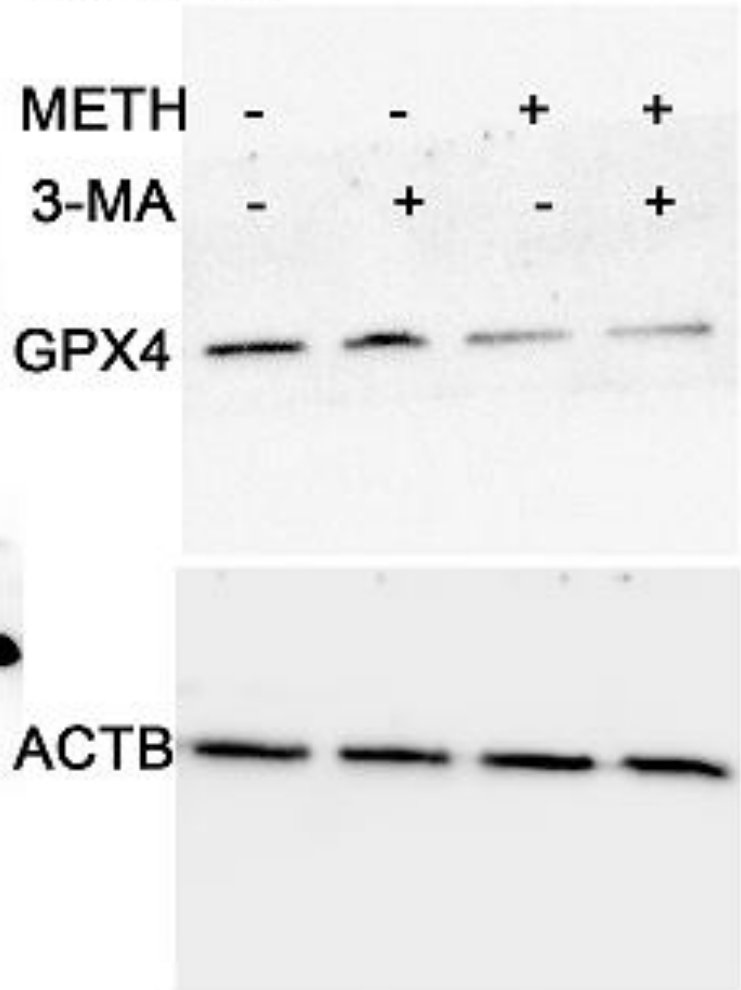


Figure 4 Q

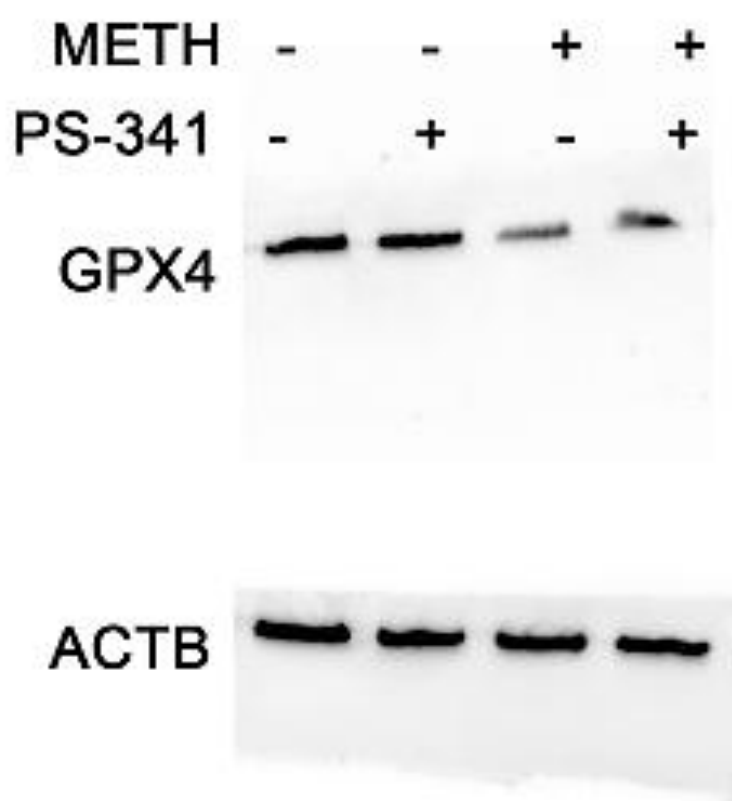


Figure 4O

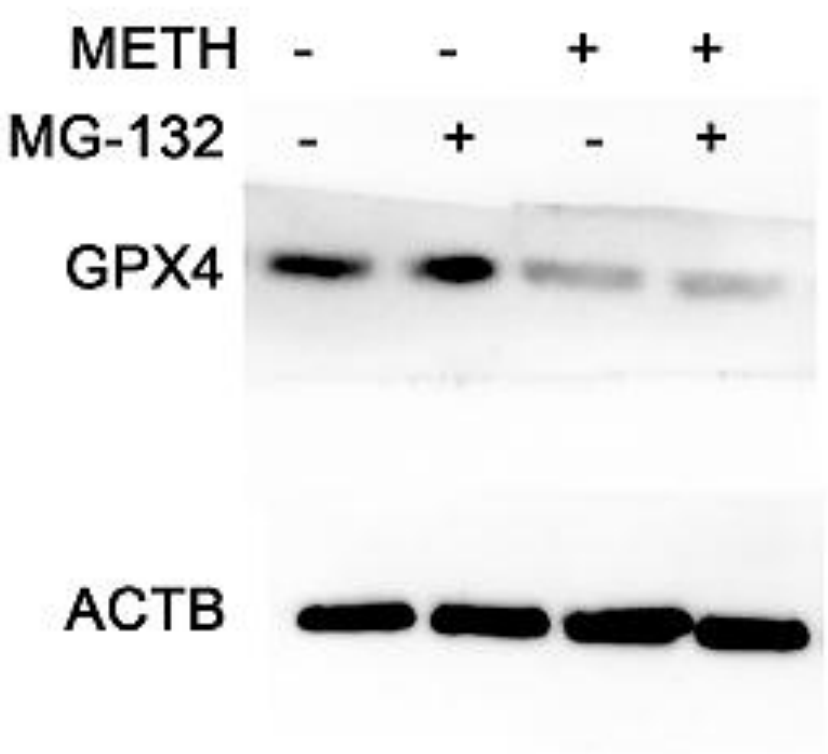


Figure 4S

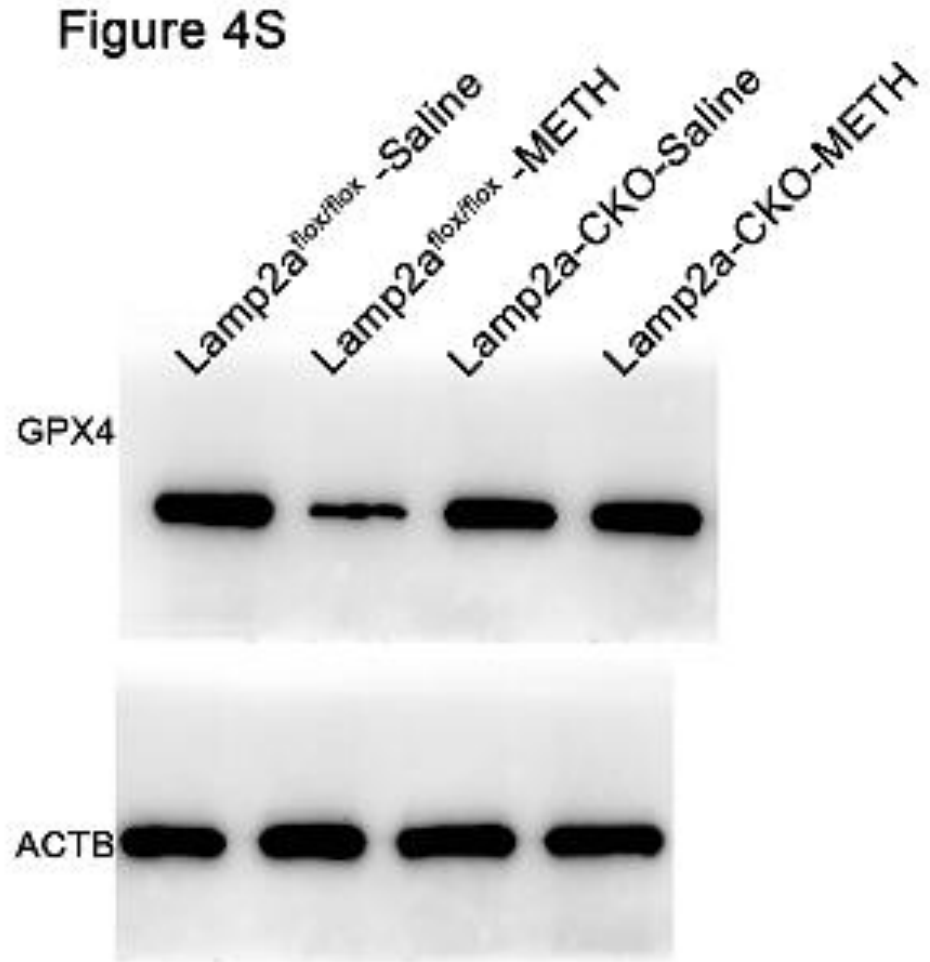

# Figure 5C

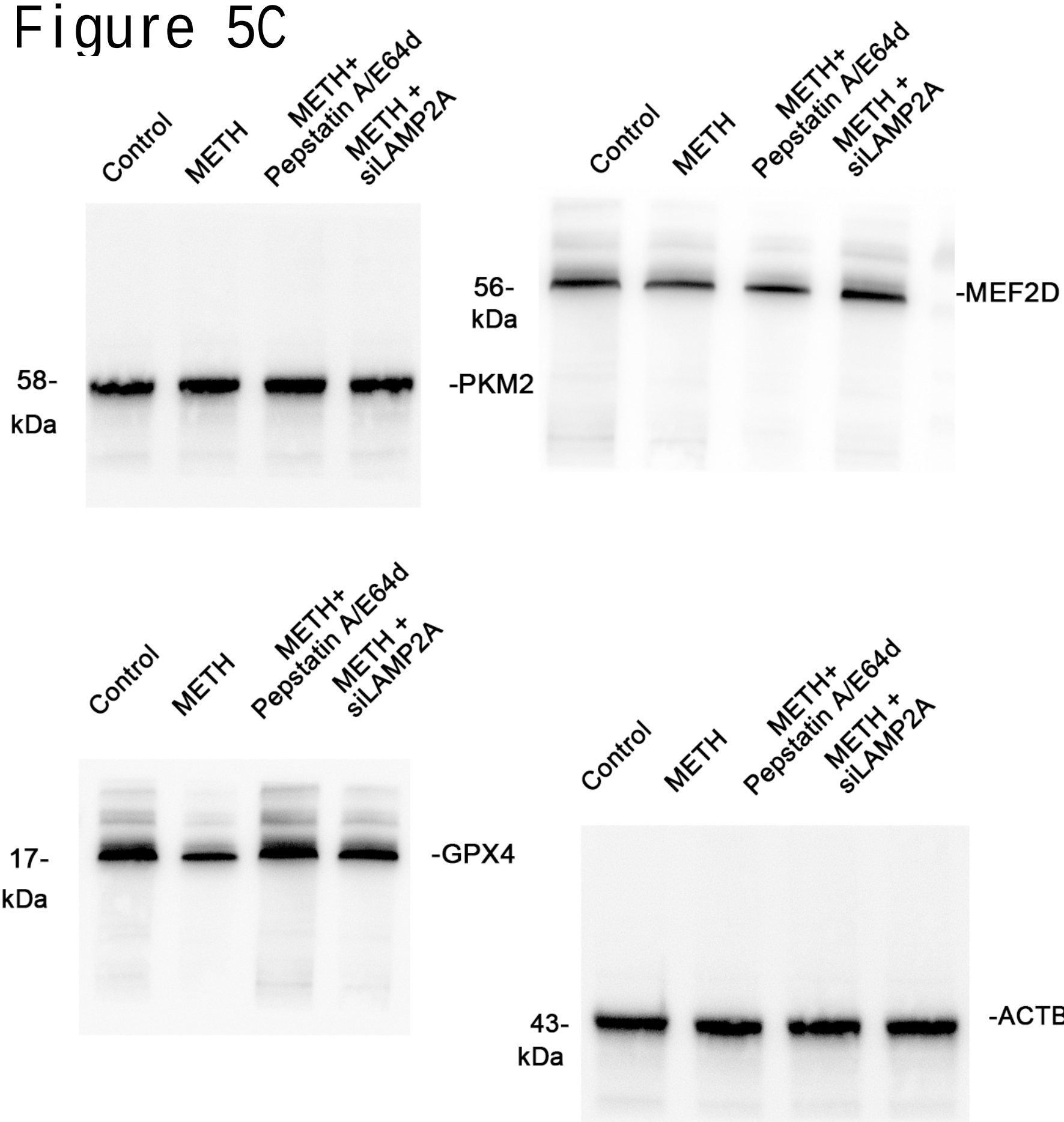

Control
METH
METH+ Pepstatin A/E64d
METH + siLAMP2A
58- kDa
-PKM2
56- kDa
-MEF2D
17- kDa
-GPX4
43- kDa
-ACTB

Figure 6C

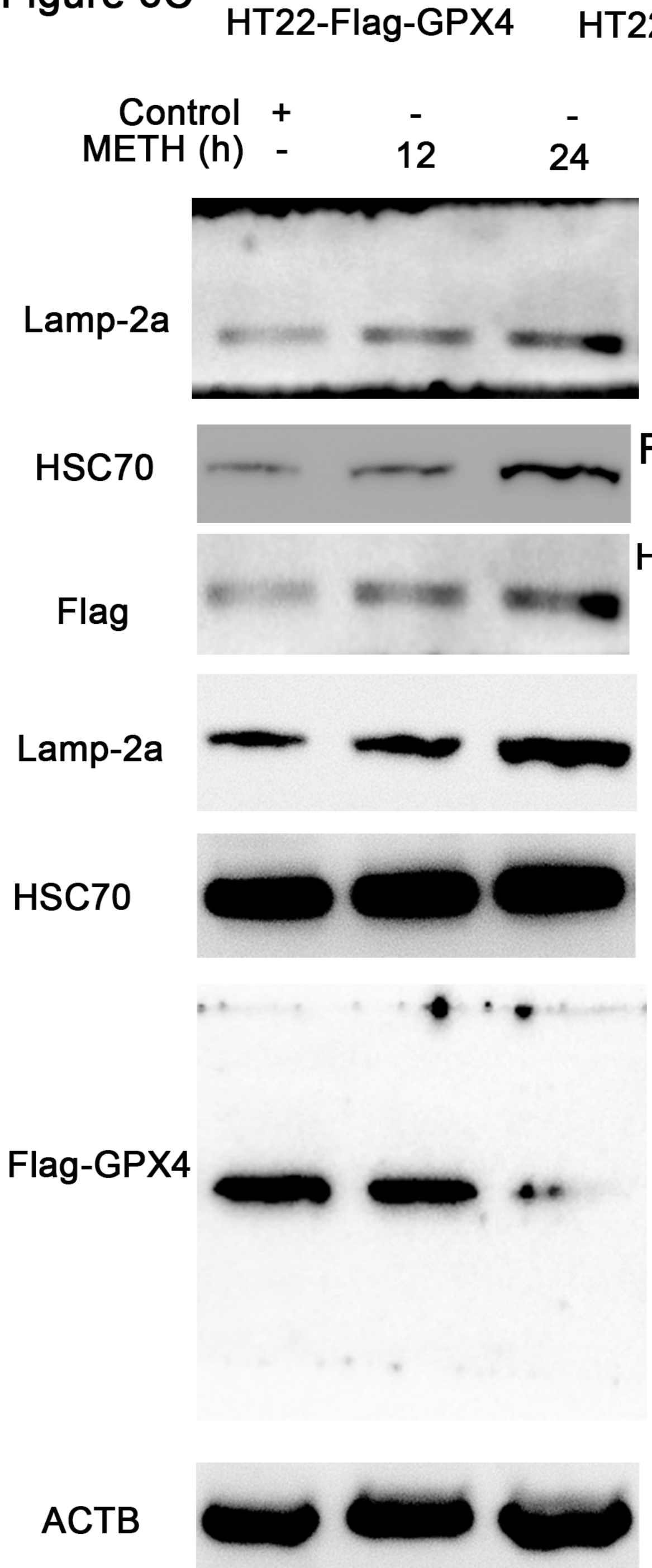


Figure 6F

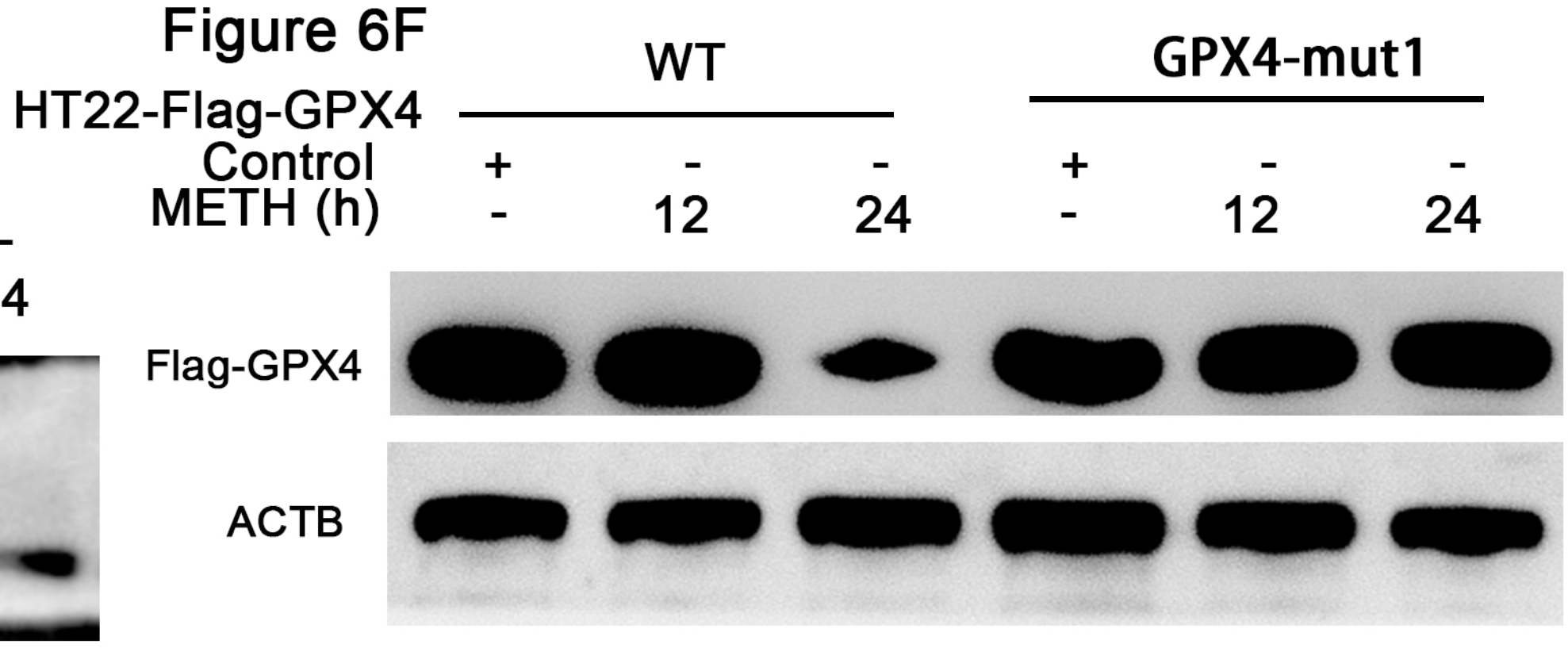


Figure 6G

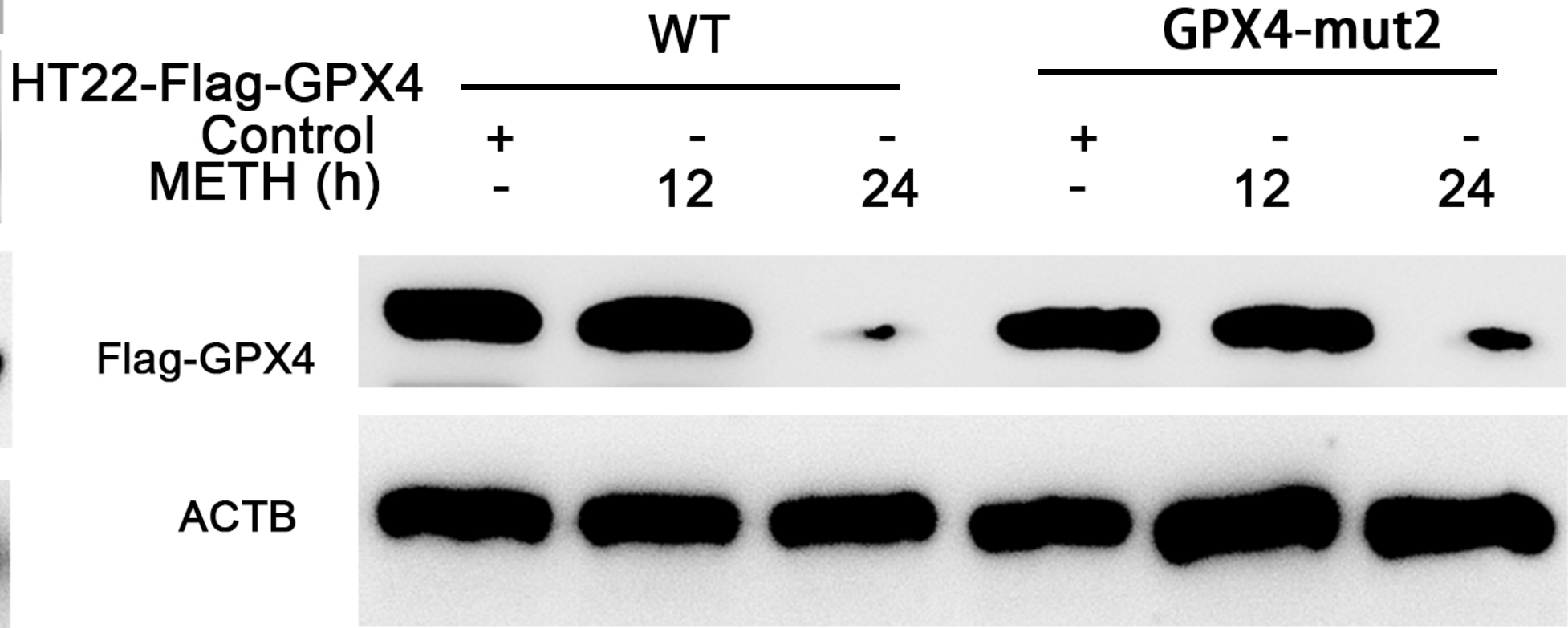


Figure 6H

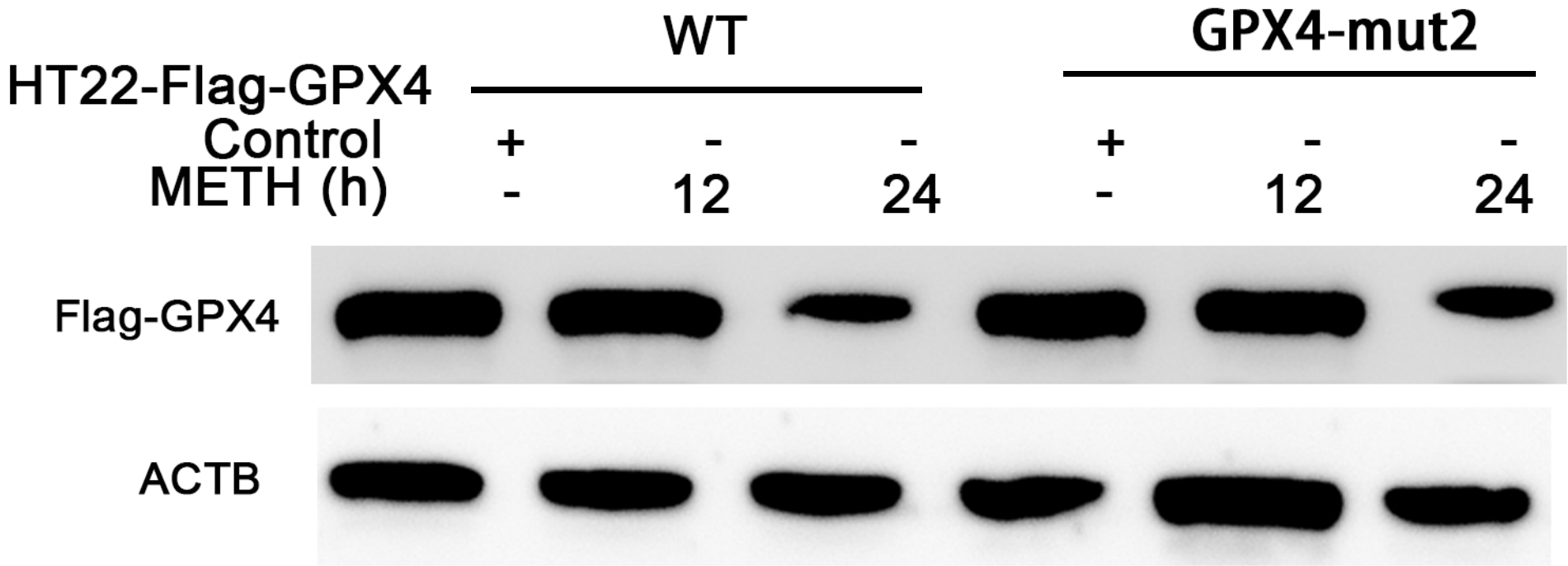


Figure 6D

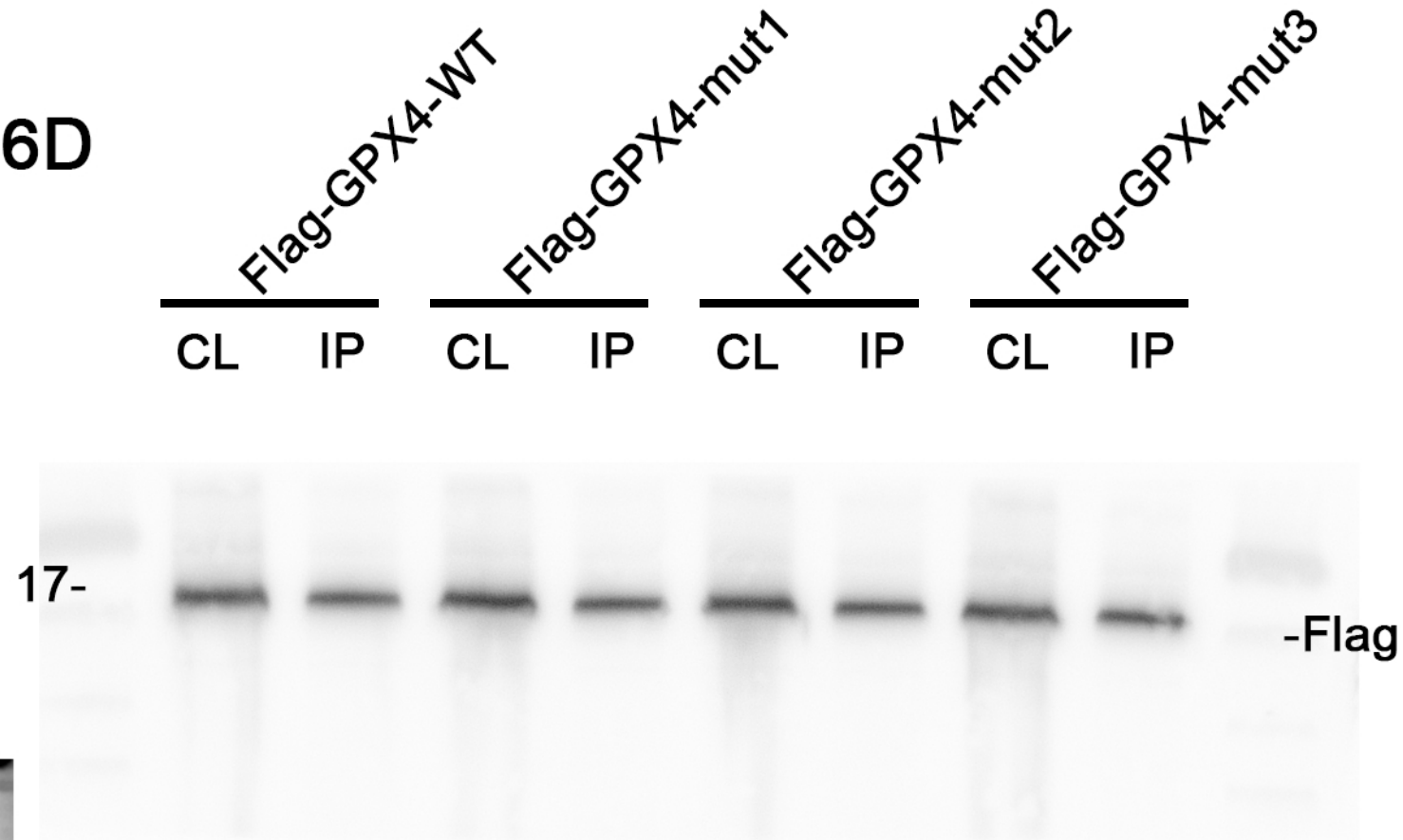


Figure 6I

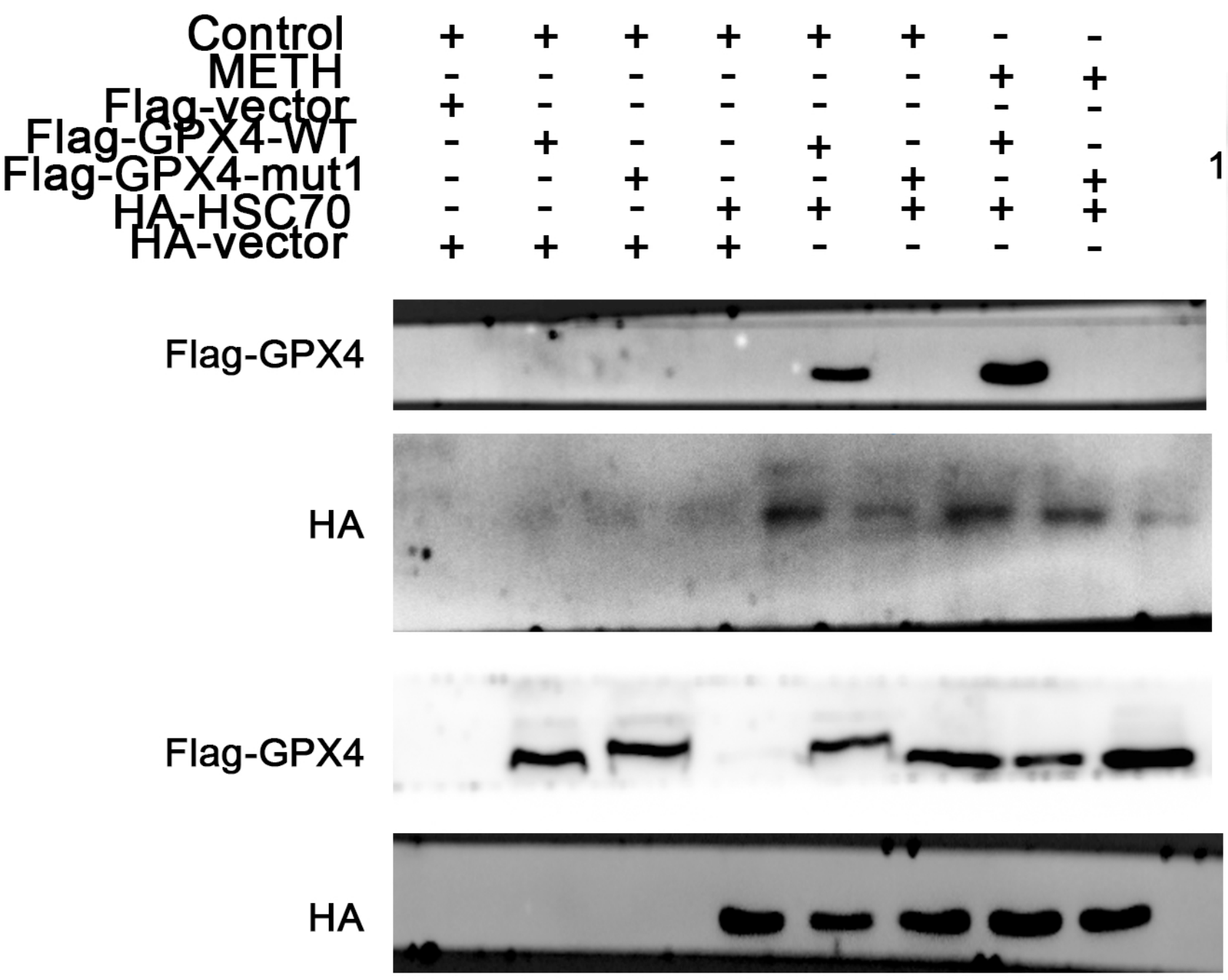

Figure S2

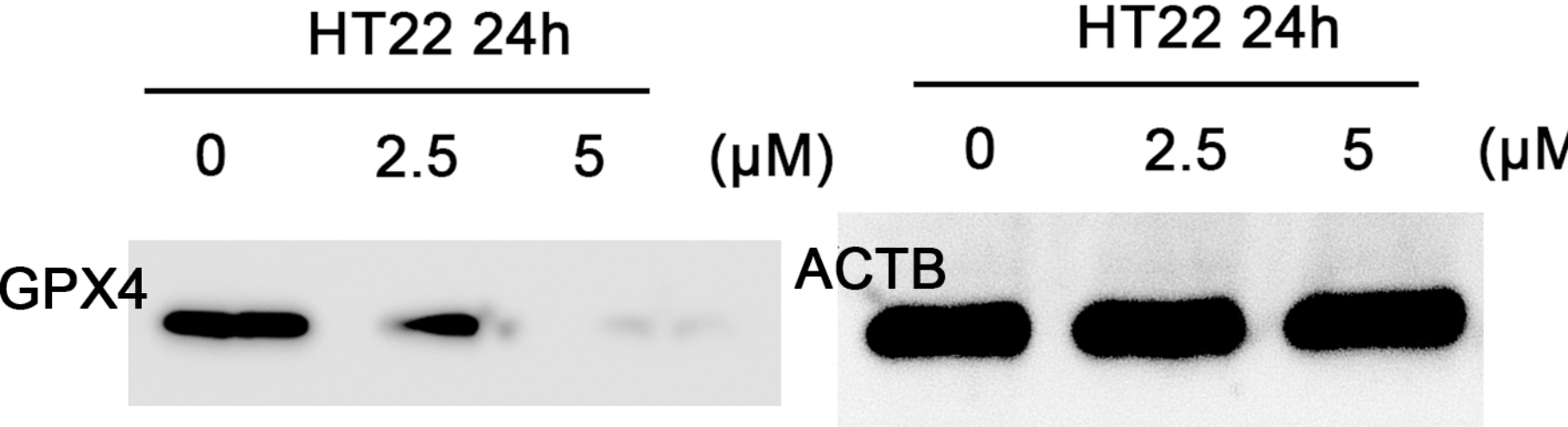


Figure S5

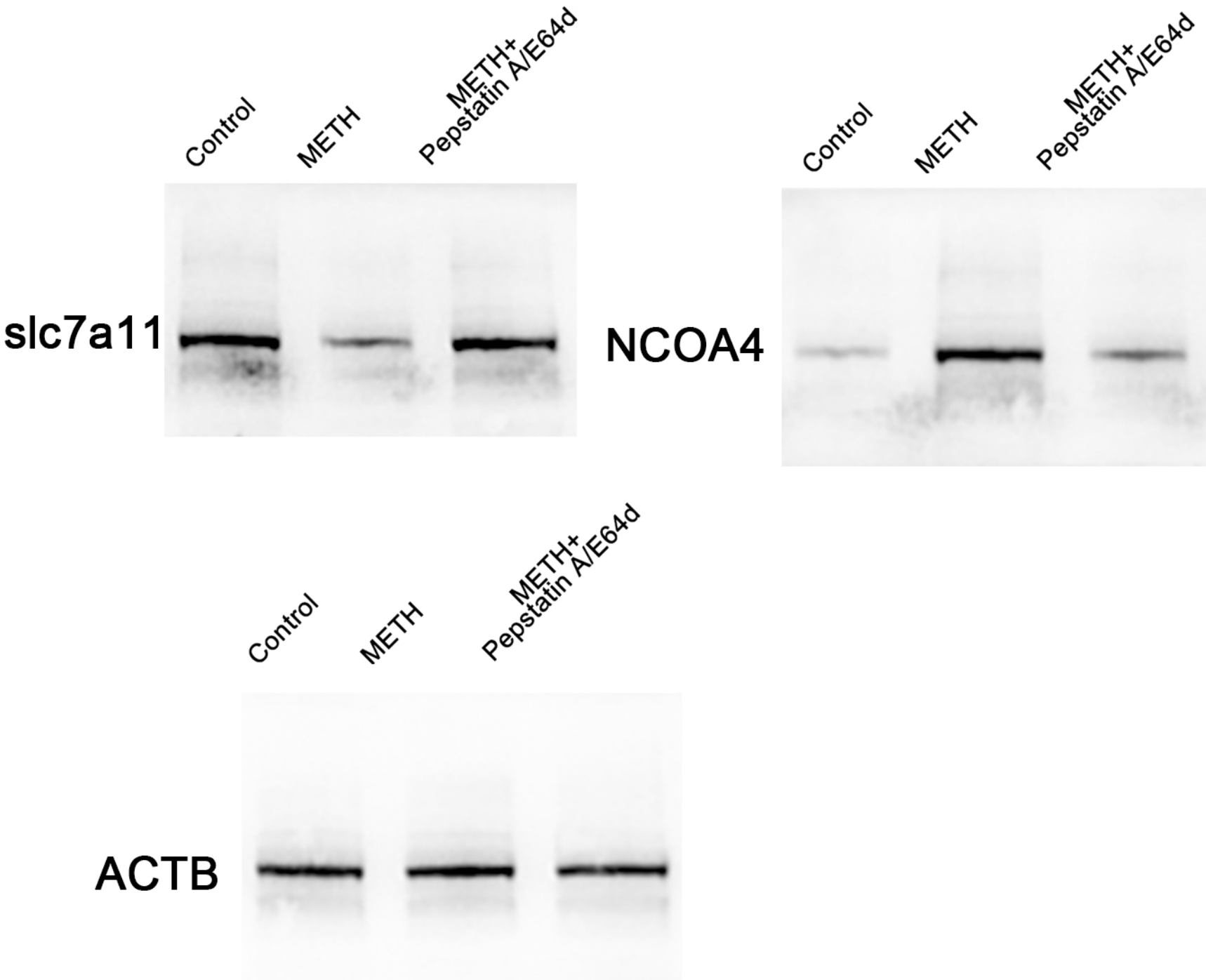

Supplement: Supplementary file 1 [file 40478_2026_2342_MOESM1_ESM.pdf]
