# Supplementary material for: Methamphetamine hijacks chaperone-mediated autophagy to degrade GPX4, driving ferroptosis-precipitated cognitive decline and addictive pathogenesis
Source: Acta Neuropathol Commun. 2026 Jun 15;14:177. doi: 10.1186/s40478-026-02342-7 (PMC13501791; doi:10.1186/s40478-026-02342-7)
Supplement: Supplementary file 2 [file 40478_2026_2342_MOESM2_ESM.pdf]

Supplementary information for

Methamphetamine Hijacks Chaperone-Mediated Autophagy to Degrade GPX4, Driving Ferroptosis-Precipitated Cognitive Decline and Addictive Pathogenesis

This file includes:

Figures. S1 to S8

Table S1

Figure. S1.

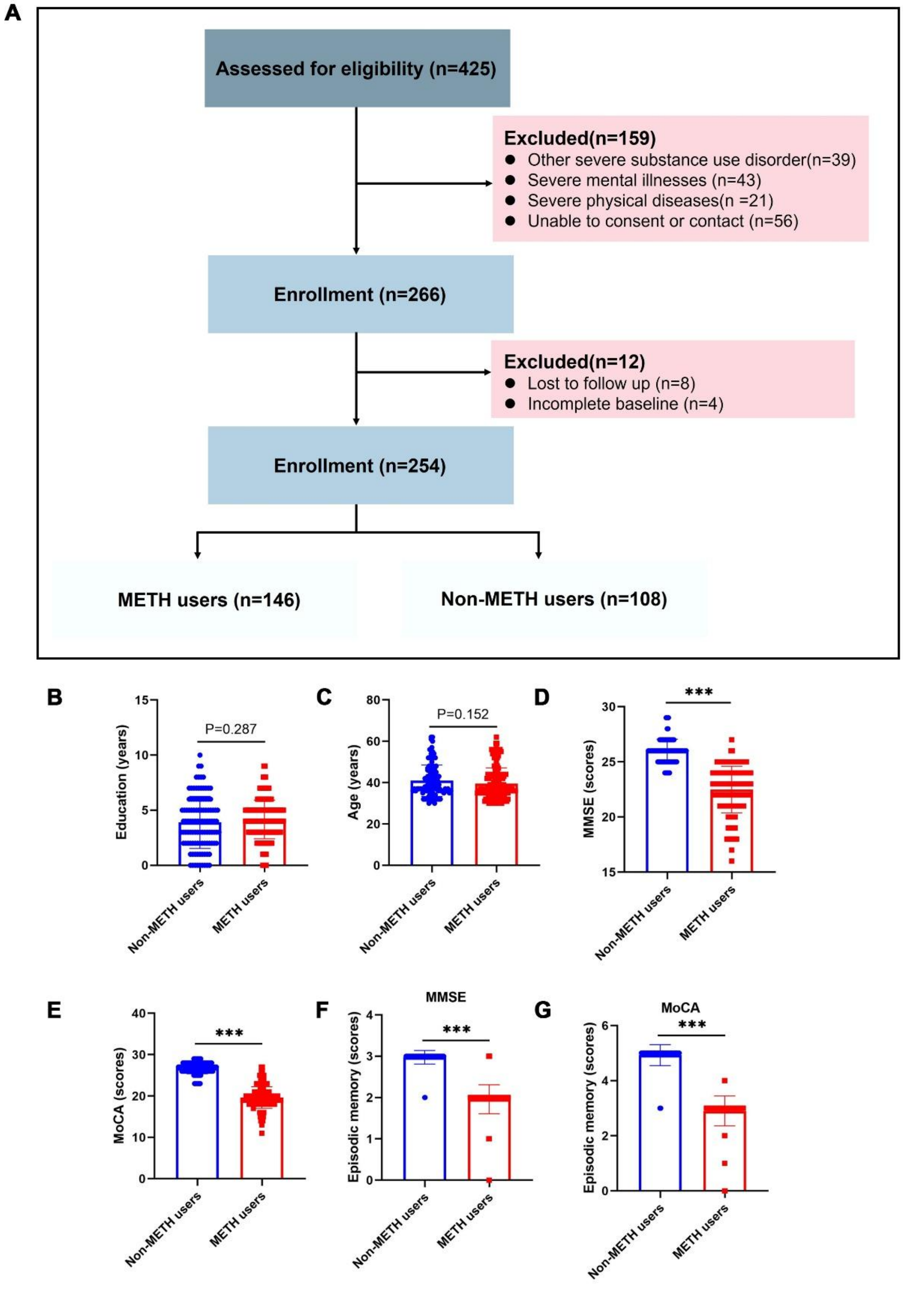
A
Assessed for eligibility (n=425)
Excluded(n=159)
Other severe substance use disorder(n=39)
Severe mental illnesses (n=43)
Severe physical diseases(n =21)
Unable to consent or contact (n=56)
Enrollment (n=266)
Excluded(n=12)
Lost to follow up (n=8)
Incomplete baseline (n=4)
Enrollment (n=254)
METH users (n=146)
Non-METH users (n=108)
B
Education (years)
P=0.287
Non-METH users
METH users
C
Age (years)
P=0.152
Non-METH users
METH users
D
MMSE (scores)
***
Non-METH users
METH users
E
MoCA (scores)
***
Non-METH users
METH users
F
MMSE
Episodic memory (scores)
***
Non-METH users
METH users
G
MoCA
Episodic memory (scores)
***
Non-METH users
METH users

Figure. S1. Consolidated Standards of Reporting Trials flowchart of participants. Bar graphs show (A) years of education, (B) age, (C) MMSE score, (D) MoCA total score, and (E) delayed verbal memory score for the Non-METH users (n=108) and METH-users (n=146). Data are presented as mean ± SEM. *$p < 0.05$, **$p < 0.01$, *$p < 0.001$(by unpaired t-test).

Figure. S2.

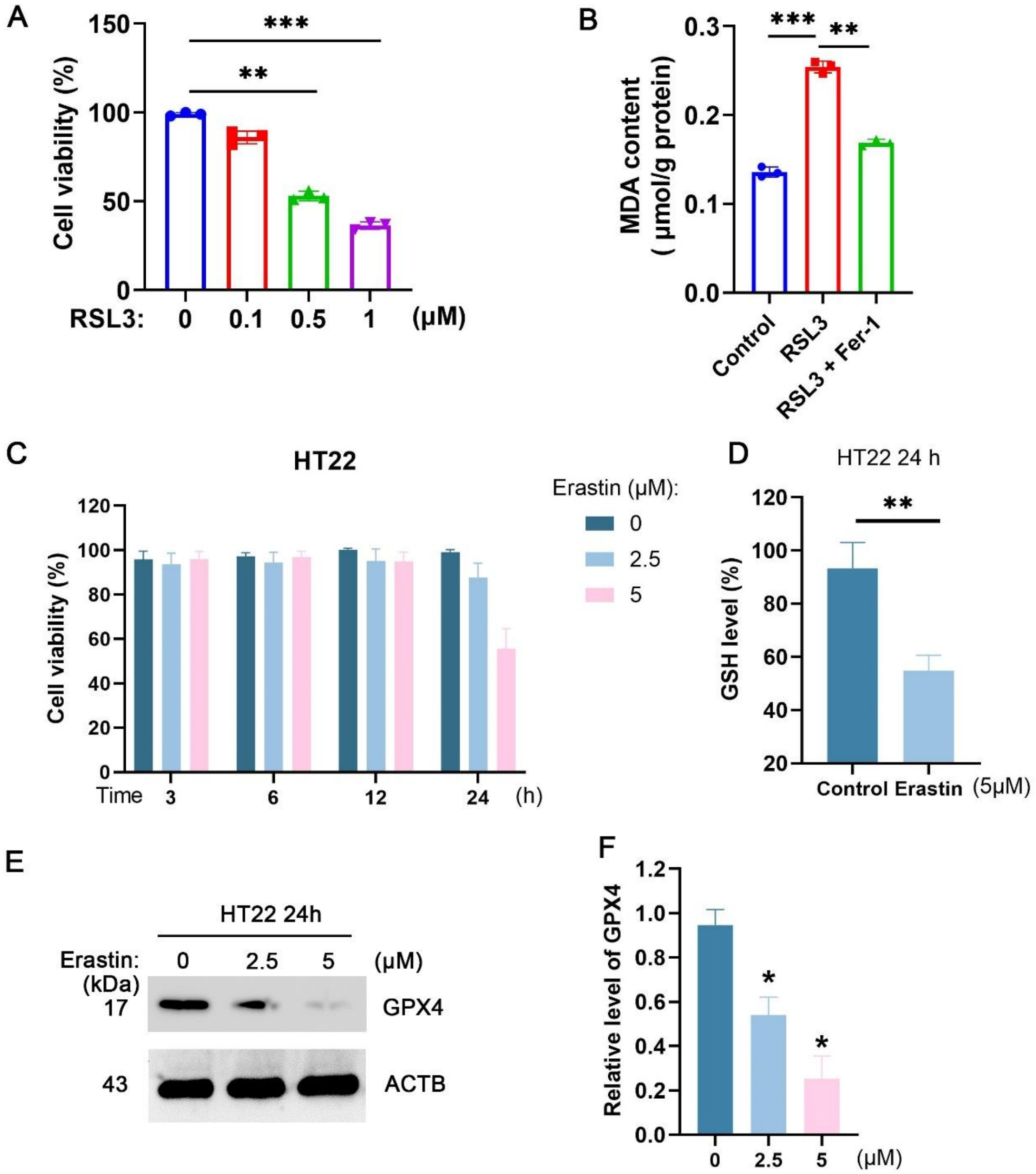

Figure S2. Hippocampal neurons are ferroptosis-sensitive cells. (A) MTS analysis of primary hippocampal neurons treated with RSL3 (0.1, 0.5 0r 1 μM) for 24 h. (B) MDA contents in primary hippocampal neurons.(C) MTS analysis of HT22 cells treated with Erastin (2.5 or 5 μM) for 3, 6, 12 and 24 h. (D) Relative GSH levels in HT22 cells treated with Erastin (5 μM) for 24 h were measured. (E) Western blotting analysis was used to detect the protein level of GPX4 in HT22 cells treated with Erastin (0, 1, 2.5 or 5 μM) for 24 h. (F) Relative GPX4 protein level. For all figures: Values mean ± SD. One-way ANOVA or two-way ANOVA. $*P < 0.05$, $**P < 0.01$, and $***P < 0.001$. All data are representative of at least three times.

Figure S3.

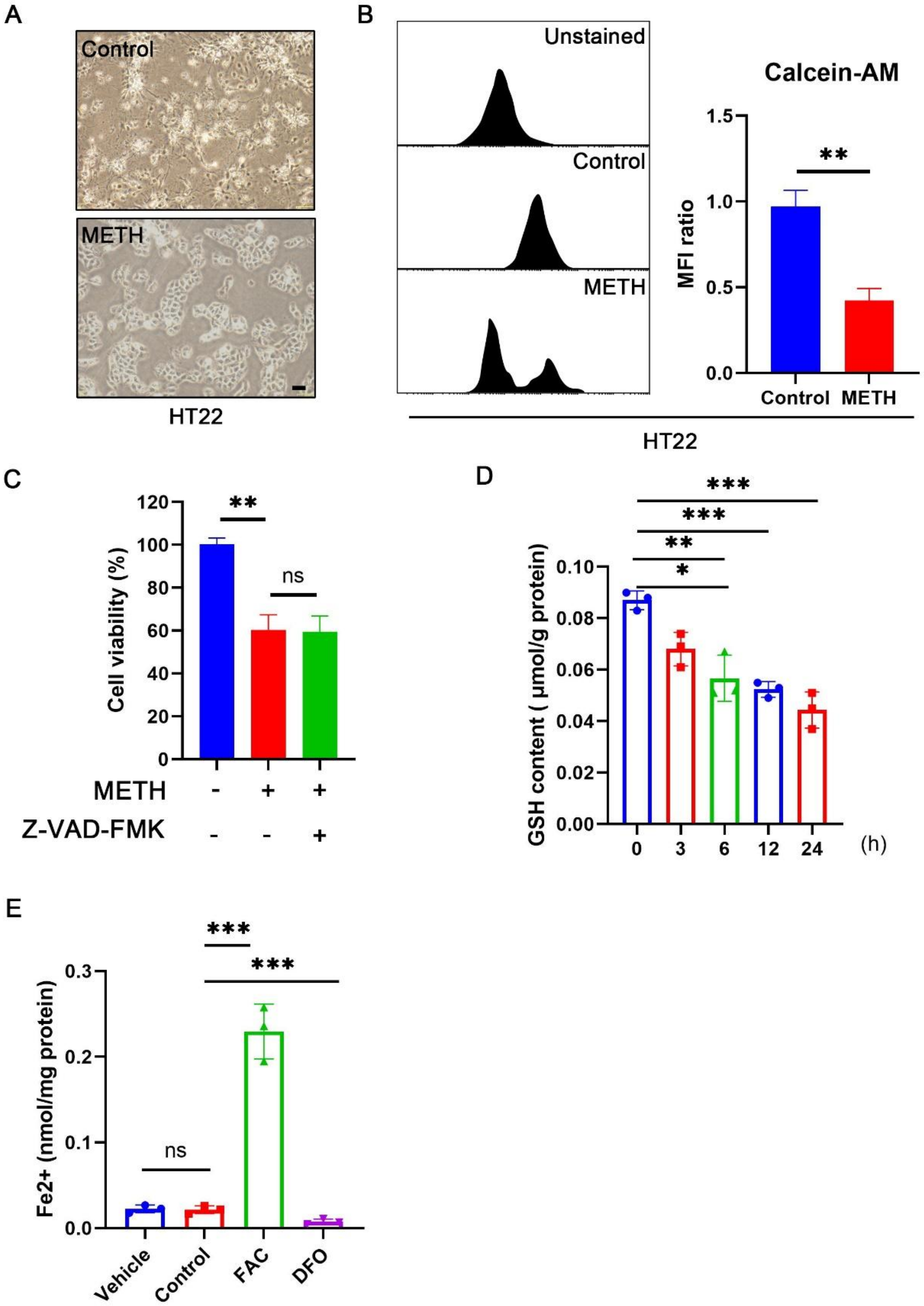

Figure S3. METH induced ferroptosis in HT22 cells. (A) Differential interference contrast microscopy images of HT22 cells treated with 2.0 mmol/L METH for 24 h. Control, untreated HT22 cells. Scale bar: 10 μm. (B) Representative FACS data for calcein AM labeling of HT22 cells treated

with 2.0 mmol/L METH for 24 h. Mean fluorescence intensity (MFI) of calcein AM was shown. (C) MTS analysis of HT22 cells following METH (2.0 mmol/L) treatment with or without Z-VAD-FMK (50 μM) for 24 h. (D) GSH levels of HT22 cells treated with METH (2.0 mmol/L) for 0, 3, 6, 12, 24 h. (E) Fe^{2+} levels of HT22 cells treated with FAC or DFO. For all figures: Values mean ± SD. One-way ANOVA or two-way ANOVA. *P < 0.05, **P < 0.01, and ***P < 0.001. All data are representative of at least three times.

Figure S4.

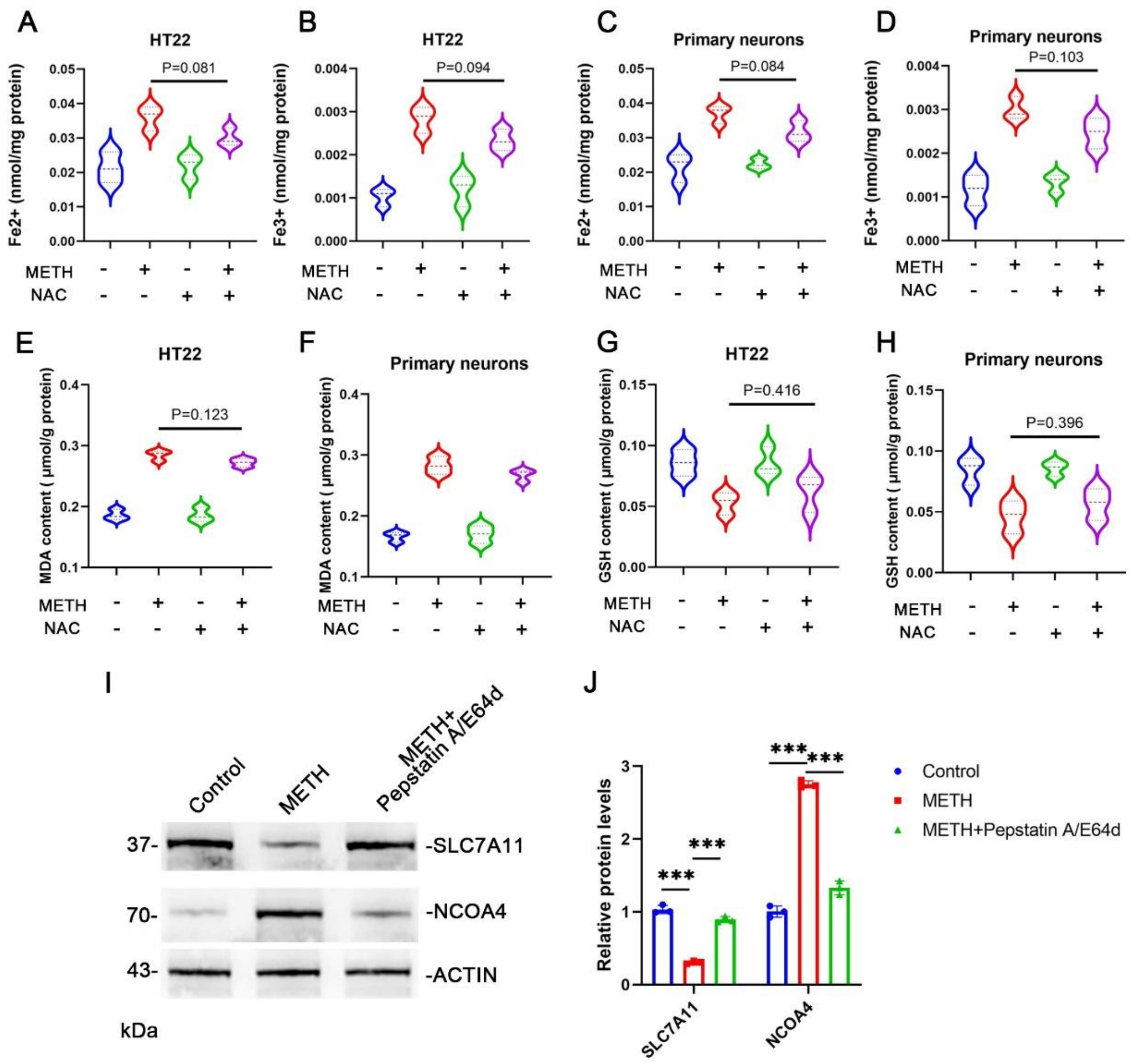

Figure S4. NAC could not reverse METH-induced lipid peroxidation and ferroptosis. (A-D) Intracellular chelatable iron levels (Fe2+ and Fe3+ levels) were determined using the Iron Assay Kit in (A, B) HT22 cells or (C, D) hippocampal-derived primary neurons following METH or NAC treatment for 24 h were determined. (E, F) MDA content in (E) HT22 cells or (F) hippocampal-derived primary neurons following METH or NAC treatment for 24 h were determined. (G, H) GSH levels in (G) HT22 cells or (H) hippocampal-derived primary neurons following METH or NAC treatment for 24 h were determined. (I) Western blotting analysis was used to detect the protein level of SLC7A11 and NCOA4 in HT22 cells treated with METH or METH+Pepatatin A/E64D. ACTIN refers to β-actin. (J) Relative protein levels of SLC7A11 and NCOA4. For all figures: Values mean ± SD. All data are representative of at least three times. One-way ANOVA or two-way ANOVA. *$P < 0.05$, **$P < 0.01$, and ***$P < 0.001$.

Figure S5.

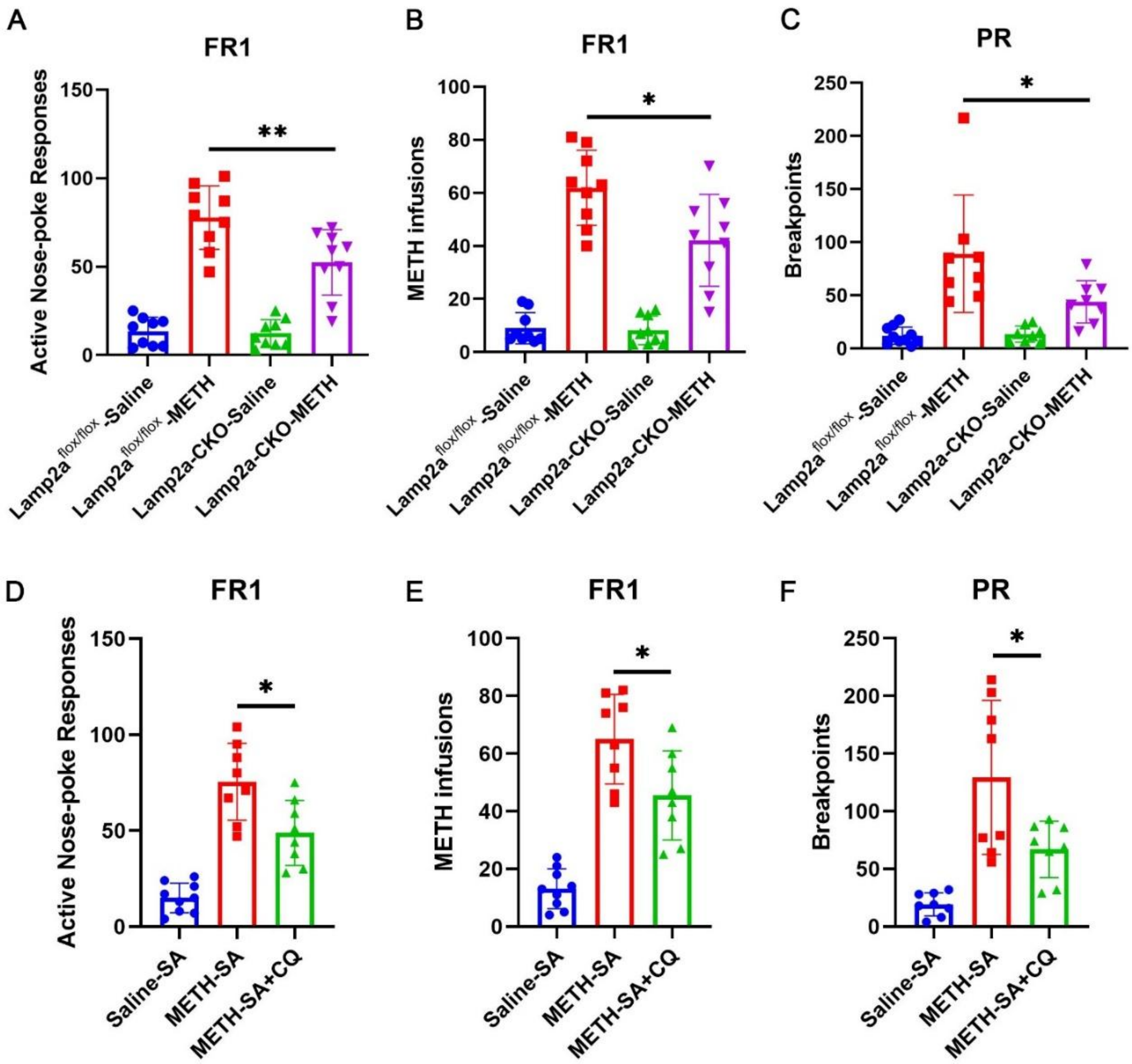

Figure S5. METH consumption and motivation were decreased by CMA inhibition. (A, D) METH-reinforced active nose-pokes and (B, E) METH-reinforced infusions under FR1 reinforcement. n=8 mice/group. (C, F) The breakpoint number under PR reinforcement. n=8 mice/group. For all figures: Values mean ± SD. n=8 mice/group. One-way ANOVA or two-way ANOVA. *P < 0.05, **P < 0.01, and ***P < 0.001.

Figure S6.

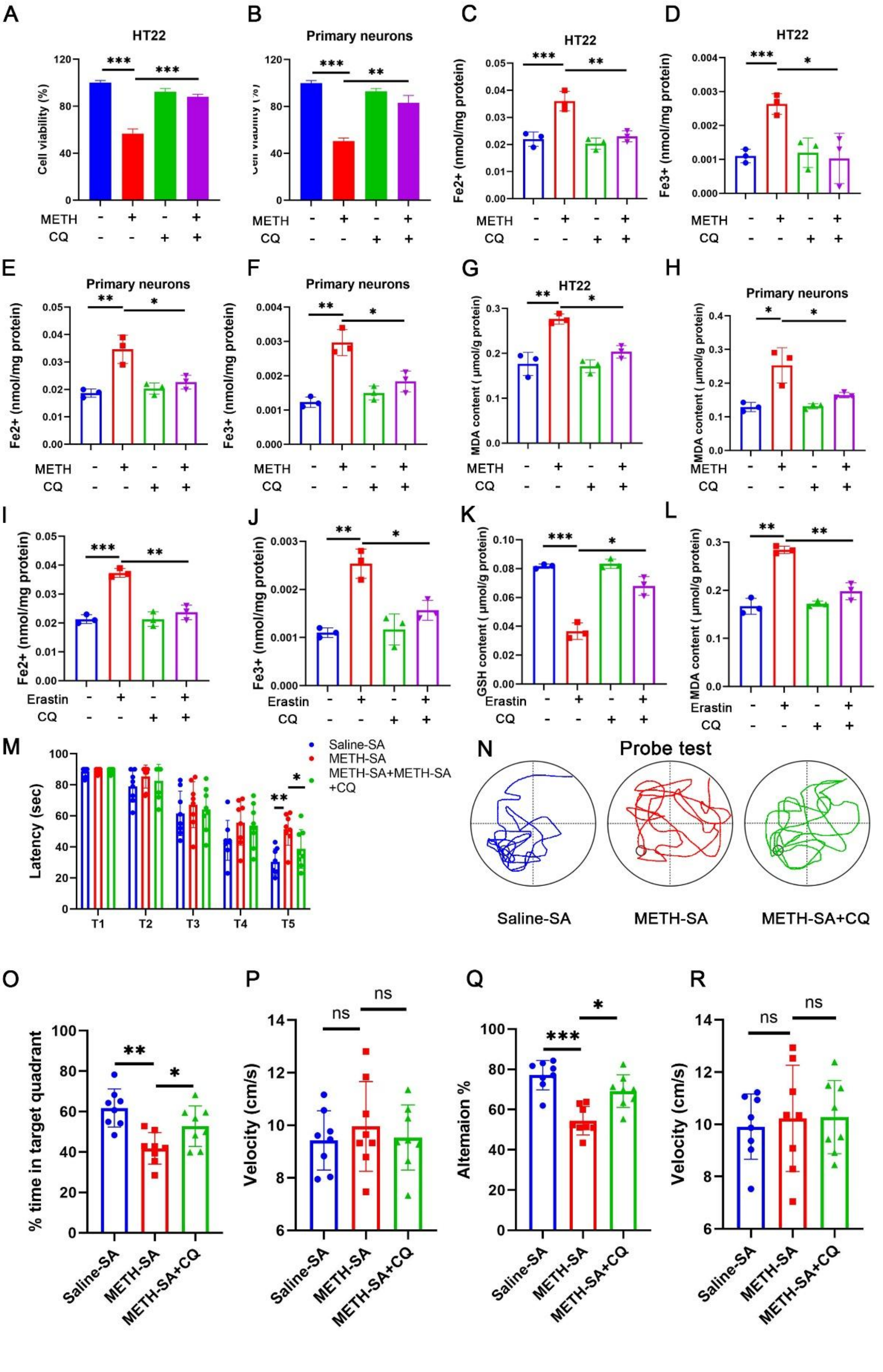

Figure S6. Inhibitor of CMA ameliorated METH-induced ferroptosis and cognitive decline.

MTS analysis of cell viability in (A) HT22 cells or (B) hippocampal-derived primary neurons following METH (2 or 1 mmol/L) treatment with CQ for 24 h. Iron assay kit was used to measure the Fe^{2+} and Fe^{3+} levels in (C-D) HT22 cells or (E-F) hippocampal-derived primary neurons following METH (2 or 1 mmol/L) treatment with CQ for 24 h. MDA contents in (G) HT22 cells or (H) hippocampal-derived primary neurons following METH (2 or 1 mmol/L) treatment with CQ for 24 h. HT22 cells treated with erastin and CQ for 24 h. Iron assay kit was used to measure the Fe^{2+} (I) and Fe^{3+} (J) levels. GSH levels (K) and MDA (L) contents in HT22 cells. (M) Escape latencies (s) of saline-SA, METH SA, and METH SA + CQ mice in the MWM test. (N) Swim paths, (O) percentage of time spent swimming in the target quadrant, and (P) average velocity during the probe test for mice in the MWM test. (Q) Alternation of arms in the spontaneous alternation test of mice in the Y-maze test and (R) average velocity during the spontaneous alternation test of mice in the Y-maze test. For all figures: Values mean ± SD. N = 8 mice/group. One-way ANOVA or two-way ANOVA. *$P < 0.05$, **$P < 0.01$, and ***$P < 0.001$. All data are representative of at least three times.

Figure S7.

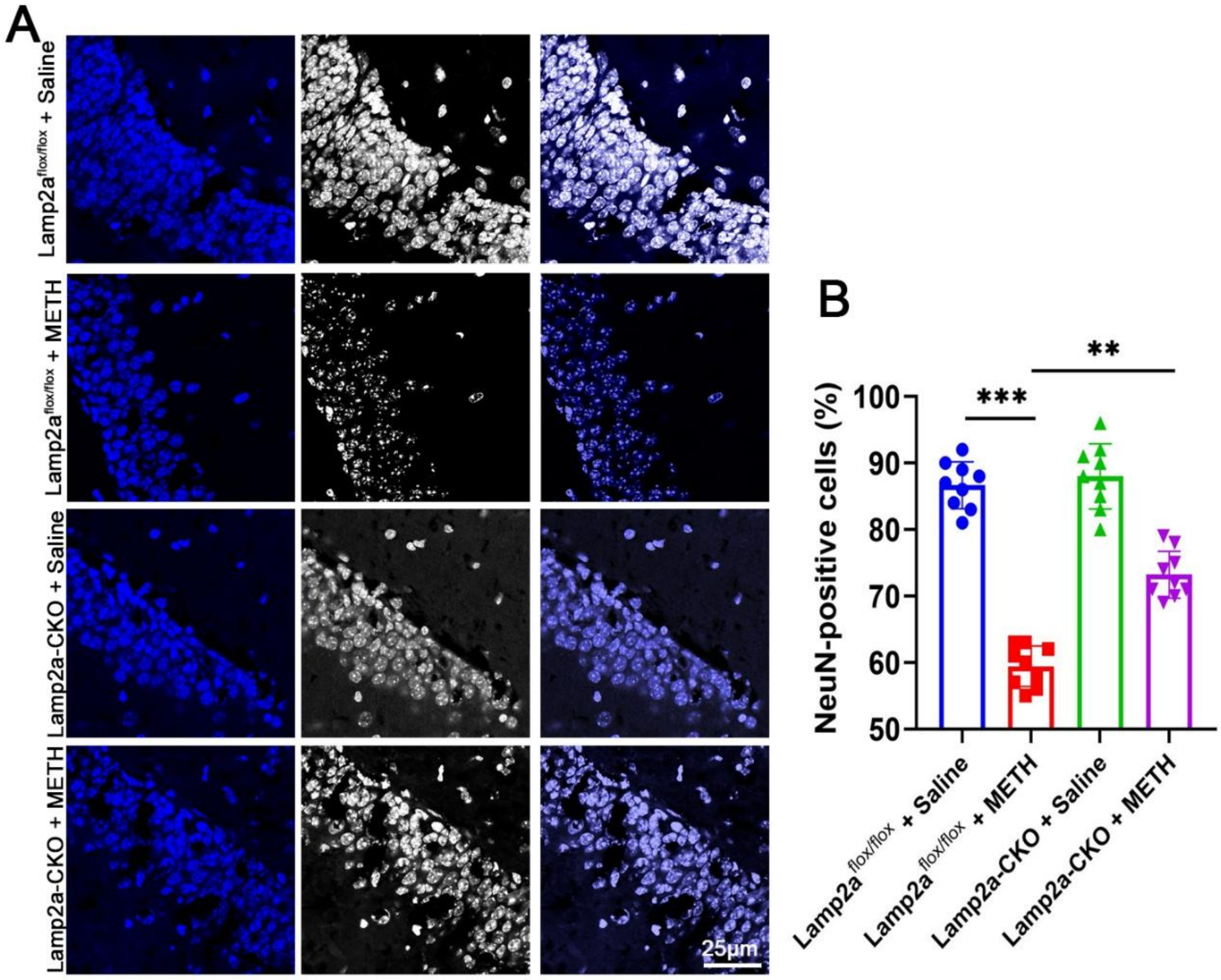

Fig. S7. Effects of Lamp2a deficiency on METH-induced neuronal loss. (A) Representative immunofluorescence images of NeuN (white) and DAPI (blue) staining in the hippocampus of Lamp2a$^{flox/flox}$ and Lamp2a-CKO mice treated with saline or METH. Scale bar = 25 μm. (B) Quantification of NeuN-positive cells expressed as a percentage of total cells (NeuN^{+}/DAPI^{+}). Data are presented as mean ± SD. **P < 0.01, ***P < 0.001.

Figure S8.

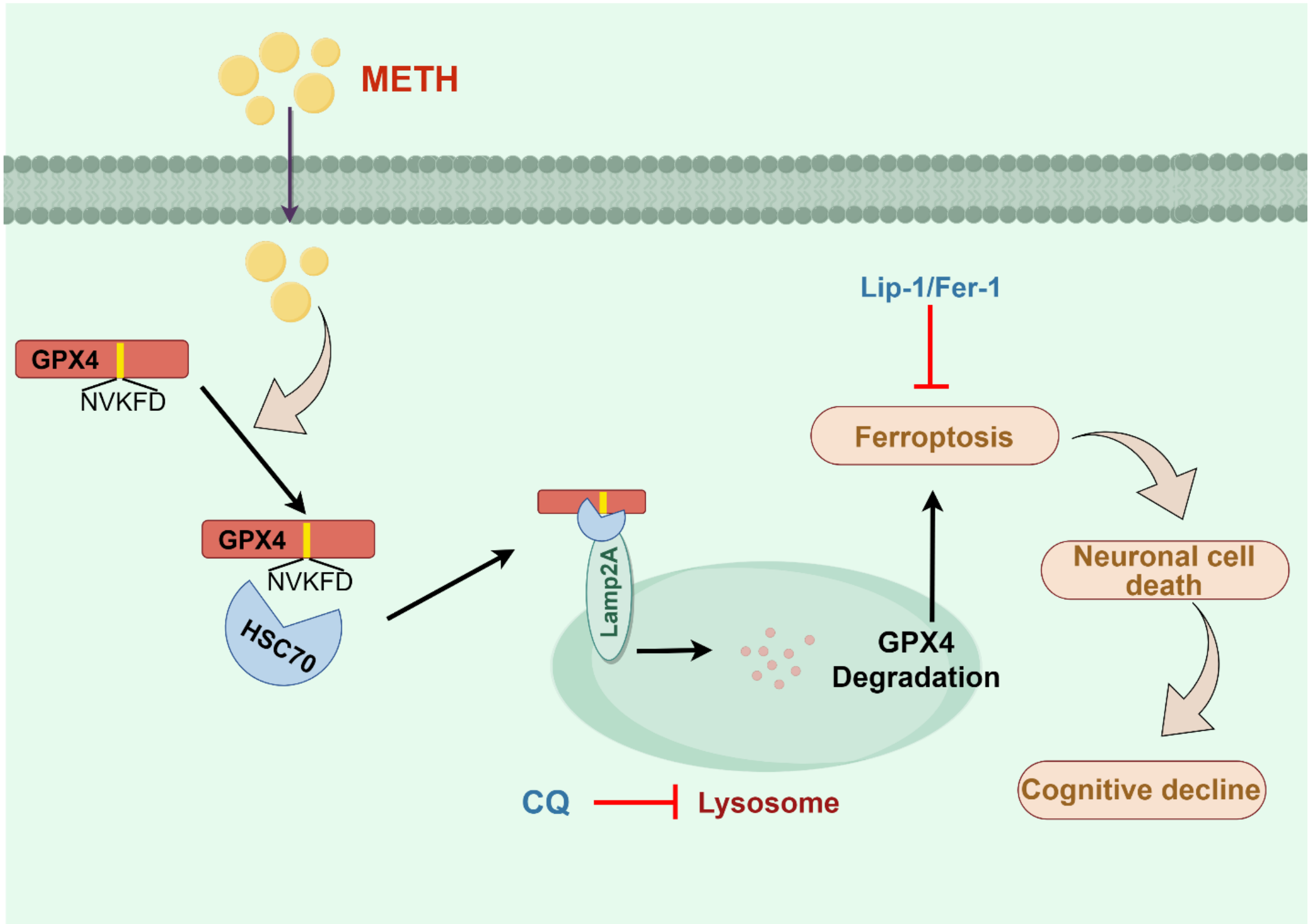

Fig. S8. Methamphetamine promotes neuronal ferroptosis through chaperone-mediated autophagic degradation of GPX4. Our study demonstrated a direct impact of CMA on the METH-induced ferroptosis of hippocampal neurons and cognitive decline in humans and mice and provided evidence of the potential value of detraction of CMA activity as a therapeutic strategy for METH-induced cognitive impairment and addiction.

Table S1:

Compound (Abbr.)	Target / Purpose	Supplier & Catalog #	Final Working Concentration	Pretreatment Duration (prior to METH)	Co-treatment with METH
N-Acetylcysteine (NAC)	Antioxidant / GSH precursor	MedChemExpress, HY-B0215	100 μM	1 h	Yes
Chloroquine (CQ)	Lysosomal inhibitor / Autophagy flux blocker	MedChemExpress, HY-17589A	25 μM	1 h	Yes
3-Methyladenine (3-MA)	Class III PI3K inhibitor / Autophagy initiation blocker	MedChemExpress, HY-19312	25 μM	1 h	Yes
MG-132	Proteasome inhibitor	MedChemExpress, HY-13259	10 μM	1 h	Yes
E64d & Pepstatin A	Cysteine & Aspartic protease inhibitors / CMA inhibitor cocktail	Santa Cruz, sc-201280B; Merck, P5318	10 μM each	1 h	Yes
PS-341 (Bortezomib)	Proteasome inhibitor (clinical grade)	MedChemExpress, HY-10227	500 nM	1 h	Yes
RSL3	GPX4 inhibition	MedChemExpress, HY-100218A	0.5 μM	1 h	NO

Table S1. Pharmacological inhibitors used in vitro studies. For inhibitor studies, HT-22 cells were pretreated with inhibitors for 1 hour prior to METH exposure. This pretreatment protocol was chosen to ensure effective pathway inhibition before the onset of METH-induced stress, allowing for the assessment of the preventive role of ferroptosis and CMA in initiating cytotoxicity.
